# Nanoparticle‐Functionalized Cellulose Through Biosynthesis‐Only Approach

**DOI:** 10.1002/advs.202511965

**Published:** 2025-09-12

**Authors:** Chunyu Ji, Ting Wang, Yifeng Wang, Qian Ding, Han Yang

**Affiliations:** ^1^ School of Chemical Sciences University of Chinese Academy of Sciences Beijing 100049 P. R. China; ^2^ School of Chemical Engineering University of Chinese Academy of Sciences Beijing 100049 P. R. China

**Keywords:** bacterial cellulose, biosynthesis, carbon dots, fluorescence, nanoparticles

## Abstract

Bacterial cellulose (BC) is a natural polymer produced by microorganisms, while carbon dots (CDs) are carbon‐based nanoparticles with biocompatibility and unique optical properties. Herein, nanoparticles, glucose‐modified CDs (Glu‐CDs), into cellulose chains using only microbial fermentation is introduced. This is the first time that nanoparticles, other than small molecules, have been biochemically incorporated into cellulose chains by forming covalent bonds in BC synthases. This biosynthesis method overcomes drawbacks associated with traditional physical and/or chemical synthetic methods, such as the easy detachment of functional groups and environmentally harmful processes. The resulting Glu‐CDs functionalized BC (Glu‐CDs‐BC) exhibits distinctive cyan fluorescence with notable changes in the micro‐structures of the pristine BC, creating plenty of protrusions on the cellulose nanofibers. Additionally, both the crystallinity and thermal stability are significantly reduced compared to the pristine BC. All these property changes cannot be achieved by simply physically attaching CDs to the BC. This study demonstrates that cellulose can be in situ functionalized through a microbial synthesis system with nanoparticles, opening up possibilities for creating functional nanoparticle‐living organism composites through solely microbial biosynthesis.

## Graphical abstract

This study reports the first covalent incorporation of glucose‐modified carbon dots (Glu‐CDs) into bacterial cellulose (BC) via microbial fermentation, resulting in BC nanofibers that exhibit uniform cyan fluorescence. A 3D‐printable hydrogel (Glu‐CDs‐BC‐SA) is developed, facilitating the fabrication of fluorescent structures. This work demonstrates an eco‐friendly approach to creating covalently incorporated nanoparticle‐cellulose composites through a purely microbial biosynthesis process.

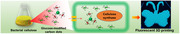

## 1 Introduction

The widespread use of petroleum‐based products has posed significant environmental challenges, underscoring the urgent need for the development of sustainable materials to reduce reliance on petroleum‐based resources.^[^ ^1^ ^]^ Cellulose is the most abundant natural polymer on Earth, constituting a polysaccharide formed by the linear linkage of D‐glucose through 1,4‐β‐glycosidic bonds. Cellulose can be obtained from various sources, such as plants, algae, tunicates, and even some species of bacteria.^[^ ^2^, ^3^ ^]^ BC is synthesized from glucose molecules through biosynthetic approaches, boasting unique advantages including exceptional purity (completely excluding lignin, hemicellulose, and pectin), high crystallinity (80%–90%), and biocompatibility. These unique properties and inherent advantages position BC as a versatile material suitable for numerous applications, including in the medical, environmental, food, and industrial sectors.^[^ ^4^, ^5^, ^6^, ^7^ ^]^ The optimization of BC's performance is typically achieved through physical or chemical methods.^[^ ^8^, ^9^, ^10^ ^]^ Physical methods offer notable advantages, such as mild modification conditions and operational simplicity. However, the weak interaction forces associated with physical methods lead to the easy detachment of loaded functional molecules or particles, potentially compromising the performance of composite materials. Although functional materials obtained through chemical modification exhibit stable performance, chemical modification strategies involve the use of strong acids, bases, and organic reagents, thereby raising concerns about potential environmental problems. Additionally, owing to the inherent poor solubility of cellulose, the efficiency of chemical modification is usually hindered.

The functionalization of BC through microbial fermentation has emerged as a promising alternative technique with the potential to overcome the limitations associated with physical and chemical modification.^[^ ^11^, ^12^ ^]^ For example, Sun et al. utilized N‐acetyl‐D‐glucosamine as a substrate for the in situ biosynthesis of BC with acetyl groups, demonstrating its efficacy as filters in removing particulate matter particles from the air.^[^ ^13^ ^]^ Wu et al. used calcium gluconate as a carbon source to synthesize a BC hydrogel with carboxyl groups through bacterial fermentation, demonstrating high ionic conductivity and suitability for low‐grade heat energy harvesting.^[^ ^14^ ^]^ Gao et al. utilized 6‐carboxyfluorescein‐modified glucose to produce BC with fluorescence properties through in situ bacterial fermentation.^[^ ^15^ ^]^ Similarly, Liu et al. incorporated glucosamine‐modified aggregation‐induced emission (AIE) luminogenic into BC through in situ bacterial fermentation to obtain AIE‐active cellulose.^[^ ^16^ ^]^ Tang et al. used photosensitizers modified glucose to endow BC with excellent fluorescence and light‐triggered photodynamic bactericidal activity for skin wound repair through in situ bacterial metabolism.^[^ ^17^ ^]^ Despite these advancements in the biosynthesis of functional BC, it is noteworthy that researchers commonly utilize small‐molecule‐modified glucose as the nutrient source. Whether glucose‐modified nanoparticles can actively participate in this biosynthesis process remains an area requiring further investigation.

Carbon dots (CDs), classified as ultra‐small (<10 nm) luminescent nanomaterials, represent a notable category within carbon‐based nanoparticles. CDs consist of a carbonized “core” and a surface “shell”, exhibiting excellent fluorescence performance, ultra‐small size, rich functional groups, and high biocompatibility.^[^ ^18^ ^]^ Therefore, CDs have become a type of multifunctional and easily modifiable nanoplatform, widely used in various biological imaging, disease treatments, and biosensing.^[^ ^19^, ^20^, ^21^, ^22^, ^23^, ^24^ ^]^ The ultra‐small size of CDs facilitates their cellular entry, even enabling access to nuclei, a region that is often inaccessible to other types of nanomaterials.^[^ ^25^ ^]^ Due to their ultra‐small size, non‐toxicity, and good biocompatibility, CDs show promising potential for active involvement in the biosynthesis process.

To the best of our knowledge, this study successfully achieved the fabrication of CDs‐functionalized BC (Glu‐CDs‐BC) through a biosynthesis‐only approach for the first time. The Glu‐CDs‐BC exhibited distinctive cyan fluorescence. Transmission electron microscope (TEM) images showed that due to the incorporation of Glu‐CDs, the Glu‐CDs‐BC displayed a large number of convex structures compared to the pristine BC nanofibers. X‐ray diffraction (XRD) results showed that the crystallinity of Glu‐CDs‐BC was 79.5%, which was significantly lower than that of the pristine BC (90.3%). Additionally, the thermal analysis revealed that the thermal decomposition temperature of the Glu‐CDs‐BC (215 °C) was much lower than the pristine BC (293 °C). These findings suggest that the presence of Glu‐CDs as building blocks for glucose chains of Glu‐CDs‐BC contributes to the formation of more amorphous regions, thereby reducing both the crystallinity and the thermal decomposition temperature. Using a simple mixing process with Glu‐CDs‐BC and SA (sodium alginate), we prepared a printable composite hydrogel (Glu‐CDs‐BC‐SA), which was used to successfully print various fluorescent 3D structures, including a square frustum, hollow cuboid, and butterfly pattern. This demonstrates the capability of Glu‐CDs‐BC to construct complex 3D architectures via 3D printing, indicating its potential for a new generation of sustainable and advanced materials. The method we developed not only has great significance for the preparation of nanoparticles‐BC composite materials but also inspires advancements in the biosynthesis of composite materials based on nanomaterials and biomacromolecules using only microorganisms.

## 2 Results and Discussion

### 2.1 Biosynthesis of Glu‐CDs‐BC Using Only Microorganisms

**Figure 1:**
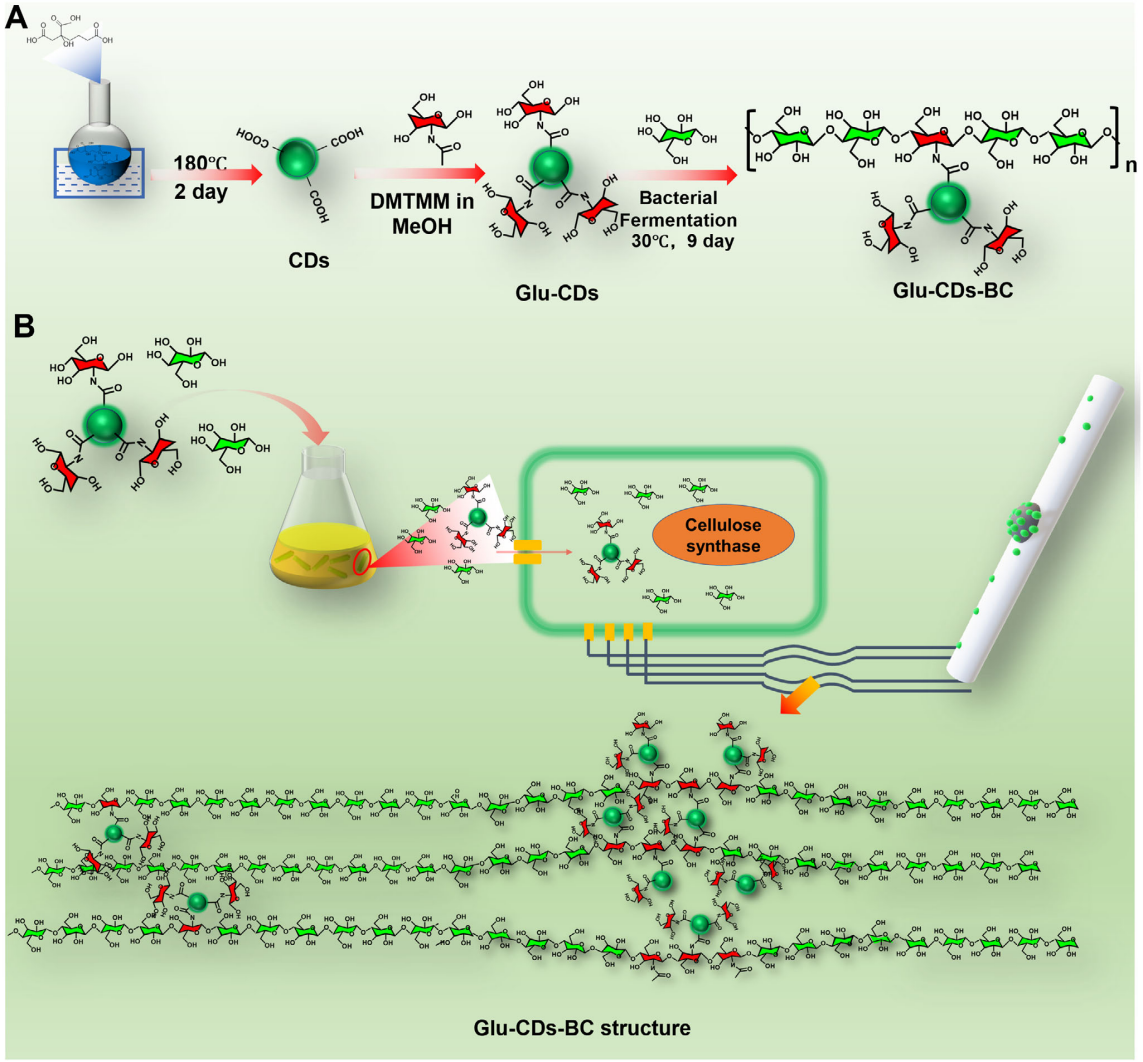
A) Schematic illustration of the preparation process of Glu‐CDs‐BC: CDs were synthesized via direct high‐temperature carbonization of citric acid and subsequently functionalized with glucose to obtain Glu‐CDs, the resulting Glu‐CDs were then added into the culture medium containing *K. sucrofermentans*, where Glu‐CDs‐BC was synthesized. B) Mechanism of Glu‐CDs incorporation: during BC biosynthesis, Glu‐CDs are covalently grafted onto the growing cellulose chains via the function of cellulose synthase.

BC is produced by the enzymatic action of the BC synthase in microorganisms.^[^ ^26^ ^]^ During fermentation, microbial metabolism of glucose leads to the production of linear β‐1,4‐glucose chains, which are subsequently secreted by bacteria into extracellular matrices to form BC pellicles.^[^ ^27^ ^]^ This unique process makes glucose an ideal carrier for functional molecules or even nanoparticles, enabling the biosynthesis of functional BC. In this study, we developed a method for preparation of CDs functionalized BC (Glu‐CDs‐BC) through biosynthesis of BC by using glucose‐modified CDs (Glu‐CDs) as one of the nutrients in the microbial synthesis process. To the best of our knowledge, there has been no previous research on the preparation of functional BC using nanoparticles (such as CDs) through a biosynthetic fermentation method. The preparation process of Glu‐CDs‐BC was illustrated in **Figure** **1**. Initially, the CDs with carboxyl groups on the surface were produced using citric acid as the carbon source through a simple heating process.^[^ ^28^, ^29^, ^30^ ^]^ Next, the surface of CDs was modified with glucose through an amide reaction to produce Glu‐CDs.^[^ ^31^, ^32^ ^]^ Finally, the synthesis of Glu‐CDs‐BC was achieved by adding Glu‐CDs into a culture medium that included a type of bacterial strains named *K. sucrofermentans*.

### 2.2 Preparation and Characterization of CDs

**Figure 2:**
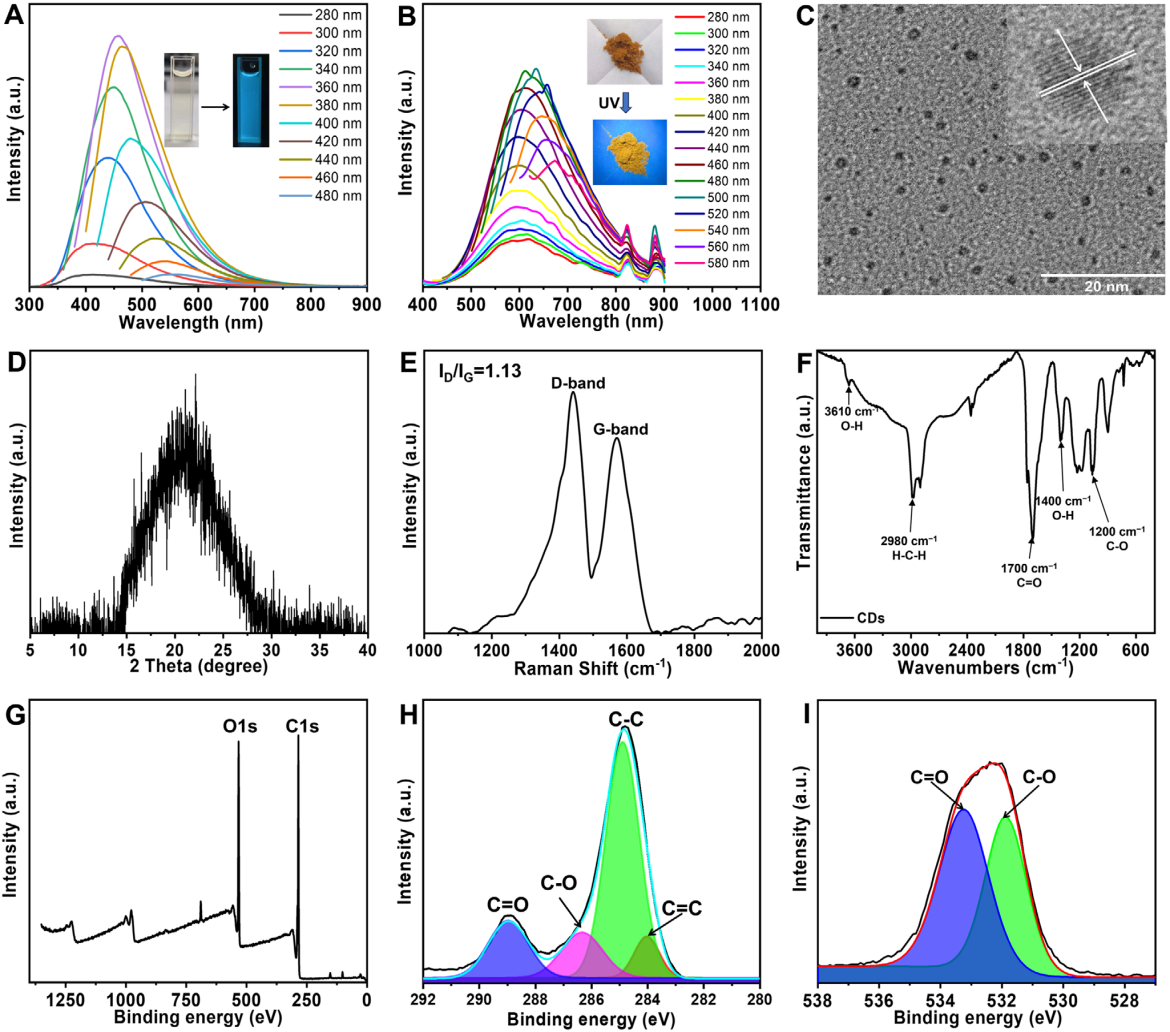
Optical, chemical, and morphological characterizations of CDs. A) PL spectra of the CDs solution excited at various wavelengths. The inset: photographs of the CDs solution under indoor light (left) and 365 nm ultraviolet (UV) light illumination (right). B) PL spectra of the CDs solid powders excited at various wavelengths. The inset: photographs of the CDs powders under indoor light (left) and 365 nm UV light illumination (right). C) TEM image of the dispersed CDs. D) The XRD pattern of the CDs. E) The Raman spectrum of the CDs. F) The FTIR spectrum of the CDs. G) The X‐ray photoelectron spectroscopy (XPS) survey spectrum of the CDs. H) The high‐resolution C1s XPS spectrum of the CDs. I) The high‐resolution O1s XPS spectrum of the CDs.

To confirm the successful preparation of Glu‐CDs‐BC through microbial biosynthesis, we first studied the morphology, optical properties, chemical compositions, and structural characteristics of the CDs, since they are crucial components in the synthesis of both Glu‐CDs and Glu‐CDs‐BC. The UV–visible absorption spectrum showed that the absorption of the CDs suspension was mainly in the UV region and extended to the near‐infrared (Figure S1, Supporting Information). The suspension of CDs was transparent and displayed blue fluorescence under UV irradiation with a wavelength of 365 nm (**Figure** **2**A, inset). As shown in Figure 2A, the fluorescence emission of the CDs suspension was significantly dependent on the excitation wavelength, as the excitation wavelength shifted from λ = 280 nm to λ = 480 nm, the peak of the emission wavelength shifted from λ = 400 nm to λ = 570 nm. This fluorescence phenomenon was mainly attributed to the uneven size distribution of the CDs ^[^ ^33^ ^]^

It was worth noting that the CDs we synthesized were resistance to self‐quenching even at the solid state. Under the excitation of UV light with a wavelength of 365 nm, the CDs powder still emitted yellow fluorescence (Figure 2B, inset), which distinguishes them from many other reported CDs, that do not display luminescence in solid‐state due to aggregation‐induced luminescence quenching ^[^ ^34^ ^]^ The main factor contributing to the solid‐state luminescence observed in our CDs is the presence of abundant uncarbonized segments on their surfaces. These uncarbonized portions lead to an expansion of inter‐nuclear distances within the CDs, preventing energy transitions. The existence of uncarbonized segments on the surface of the CDs was corroborated through the findings of thermogravimetric analysis (TGA) and differential scanning calorimetry (DSC) tests, as depicted in Figures S2 and S3 (Supporting Information). Similar to the CDs suspension, the fluorescence spectra of the CDs powder were also depended on the excitation wavelengths, and the wavelength of their emitted light was extended to the near‐infrared region of 800 nm (Figure 2B). Based on the spectral characterizations elucidated, the emission of the solid‐state CDs underwent a red shift compared to the emission of the liquid‐state CDs.

Transmission electron microscopy (TEM) image showed that the CDs we synthesized were well dispersed spherical nanoparticles without any aggregation, and the average lateral dimensions of the CDs were ≈2.53 ± 0.26 nm (Figure 2C; Figure S4, Supporting Information). As shown in Figure 2C, a high‐resolution TEM (HRTEM) image of the CDs further highlighted a high crystallinity with an in‐plane lattice spacing of 0.21 nm, corresponding to the (100) plane of graphite carbon. The dynamic light scattering (DLS) analysis showed the size of the CDs was 2.86 nm, which was slightly larger than the statistics data from TEM analysis (Figure S5, Supporting Information). Given that the DLS result was the hydrodynamic diameter of particles, it is expected that the size from DLS measurements is normally larger than the particle size obtained from TEM images.^[^ ^35^ ^]^ Zeta potential measurements showed that the Zate potential was −25.4 mV for the CDs, which was likely attributed to the carboxyl groups on their surface. These negative charges played a crucial role in the stabilizing and uniformly dispersing of the CDs in the suspension.

The crystal structure of the CDs was further studied by XRD, revealing a broad peak corresponding to the graphite (002) crystal plane at 21° (Figure 2D).^[^ ^36^ ^]^ Raman spectrum provided additional insights into the carbonaceous structure within the CDs (Figure 2E). Specifically, the peaks at wave numbers of 1422 cm^−1^ and 1548 cm^−1^ represented the presence of disordered D bands and crystalline G bands, respectively, with an intensity ratio of 1.13 for I_D_/I_G_.^[^ ^37^ ^]^ This indicated that the carbon in the CDs predominantly exhibited an amorphous configuration.

The functional groups and elemental compositions of the CDs were analyzed by Fourier‐transform infrared spectroscopy (FTIR) and XPS. From the FTIR spectrum (Figure 2F), an absorption peak at 3640 cm^−1^ was ascribed to the stretching vibration of O─H, the peak at 2980 cm^−1^ was due to the vibration of C─H, additionally, the peak at 1700 cm^−1^ was attributed to C═O, while the peak at 1400 cm^−1^ arose from the in‐plane bending vibration of O─H, the presence of C─O vibration was confirmed by the peak at 1200 cm^−1^.^[^ ^24^, ^38^ ^]^ These results from FTIR confirmed that the hydroxyl and carboxyl groups were present on the surface of the CDs. The XPS spectrum showed that the CDs predominantly consisted of carbon (C) at 64.1% and oxygen (O) at 35.9% (Figure 2G). The high‐resolution C‐spectrum (Figure 2H) showed four distinct carbon peaks: C─C (284.04 eV), C─C (284.90 eV), C─O (285.92 eV), and C═O (289.01 eV). The high‐resolution O‐spectrum (Figure 2I) showed two distinct oxygen peaks: C─O (531.91 eV) and C═O (532.28 eV). These results from XPS also confirmed the presence of hydroxyl and carboxyl groups on the surface of the CDs.

### 2.3 Preparation and Characterization of Glucose Modified CDs (Glu‐CDs)

**Figure 3:**
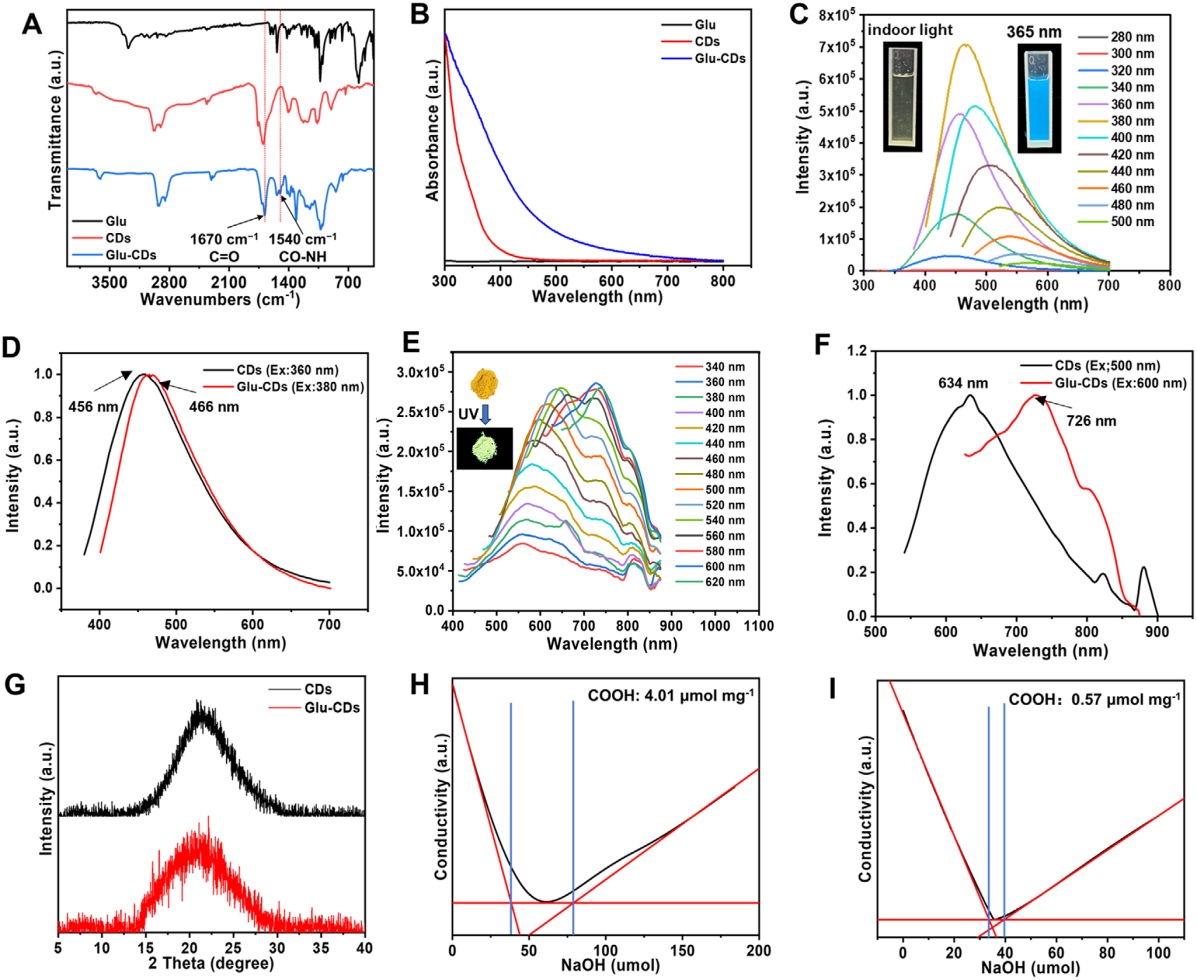
Chemical, optical and structural characterizations of Glu‐CDs. A) The FTIR spectra of the Glu‐CDs. B) The UV–vis spectra of the glucose, CDs, and Glu‐CDs. (C) PL spectra of the Glu‐CDs solution excited at various wavelengths. The inset: photographs of the Glu‐CDs solution under indoor light (left) and 365 nm UV light illumination (right). D) The optimal emission spectra of CD and Glu‐CDs solution. E) PL spectra of the Glu‐CDs solid powders excited at various wavelengths. The inset: photographs of the Glu‐CDs powders under indoor light (left) and 365 nm UV light illumination (right). F) The optimal emission spectra of CD and Glu‐CDs powders. G) The XRD patterns of the CDs and Glu‐CDs. H) Conductivity titration curve of the CDs. I) Conductivity titration curve of the Glu‐CDs.

In order to biosynthesize CDs‐modified cellulose through microbial engineering using *K. sucrofermentans*, the CDs needed to participate in the synthesis of BC and serve as a component of BC. As showed in previous studies, glucose can serve as an ideal type of carriers for functional molecules to synthesize of functional BC.^[^ ^11^, ^12^, ^13^, ^14^, ^15^, ^16^, ^17^ ^]^ Here, we tried to use glucose as the carrier for nanoparticles, therefore, we prepared glucose modified CDs (Glu‐CDs) by covalently linking glucosamine molecules onto the surface of CDs via an amide reaction between the amine groups of glucosamine and the carboxyl groups of the CDs, since the CDs have many advantages, including high biocompatibility, ultra‐small size, and excellent fluorescence. To confirm the successful modification, the Glu‐CDs were characterized by FTIR. Compared to the CDs and glucose, two new peaks formed at 1670 cm^−1^ and 1540 cm^−1^, which were corresponding to the stretching vibration of C = O and C‐N bonds of the amide bonds, respectively, indicating amide bonds were formed between the glucosamine and the CDs (**Figure** **3**A).^[^ ^11^, ^15^, ^16^, ^17^ ^]^ XPS shows that compared to CDs, Glu‐CDs contain N. The high‐resolution N spectrum of the Glu‐CDs displayed a single peak at 398.6 eV (Figure S6, Supporting Information), corresponding to the C─N signal peak, confirming the presence of the amide bond, further demonstrating the successful modification of glucose onto the surface of CDs. Compared to the CDs, UV–vis absorption spectrum showed that Glu‐CDs exhibited an enhanced light absorption between 300 nm and 800 nm (Figure 3B), while there was almost no any absorption for pure glucose. In addition, compared with the CD solution, the optimal fluorescence emission peak wavelength of Glu‐CDs solution was shifted by 10 nm (Figure 3C,D), while the optimal fluorescence emission peak wavelength of Glu‐CDs powder was shifted by 92 nm (Figure 3E,F), and this difference may due to the covalently linked glucose molecules on the surface of the CDs. The surface modification of CDs with glucose did not induce discernible alterations in their microscopic morphology or crystalline architecture (Figure 3G; Figure S7, Supporting Information). Furthermore, the carboxyl content on the surface of the CDs and Glu‐CDs was determined by conductivity titration. The results revealed that the CDs had 4.01 µmol mg^−1^ of carboxyl groups on their surface (Figure 3H), but after amide reaction with the glucosamine, the content of carboxyl groups on the Glu‐CDs was decreased to 0.57 µmol mg^−1^ (Figure 3I). This suggests that 3.44 µmol of glucose were successfully covalently linked onto the surface of each milligram of the CDs. XRD analysis can provide structure information on the core of the CDs.

### 2.4 Evidences of Glu‐CDs Participating in BC Biosynthesis

**Figure 4:**
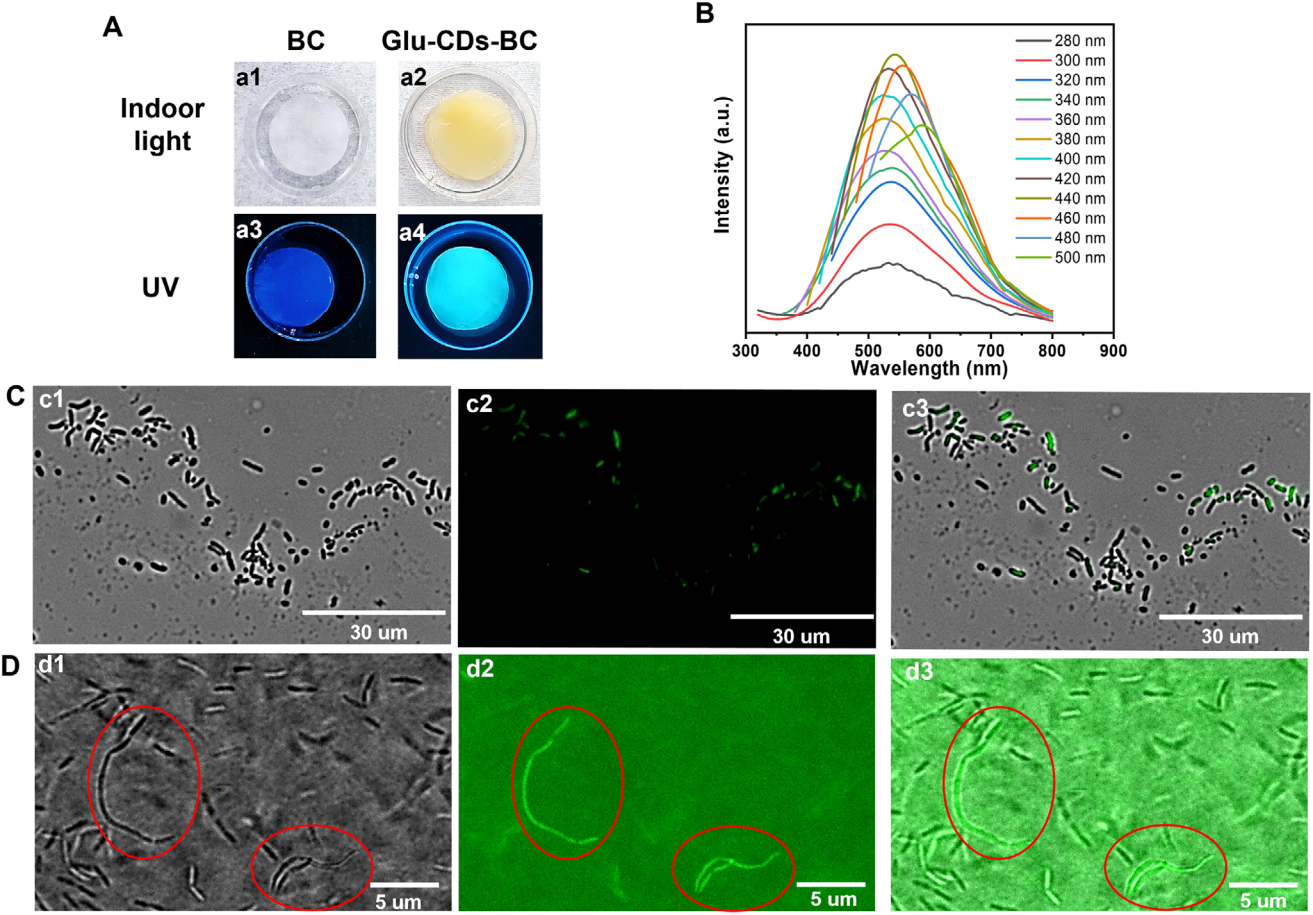
Biochemical incorporation of Glu‐CDs into BC fibers. A) Photographs of BC after purification, taken under indoor light (a1) and 365 nm UV light illumination (a3), and photographs of Glu‐CDs‐BC after purification, taken under indoor light (a2) and 365 nm UV light illumination (a4). B) Fluorescence emission spectra of Glu‐CDs‐BC after purification. C) Inverted fluorescence microscope images of *K. sucrofermentans* incubated in a culture medium containing Glu‐CDs for 24 h: (c1) Bright‐field image, (c2) Fluorescence image under blue light excitation, (c3) A merged image of the images (c1) and (c2). D) Inverted fluorescence microscope images of the Glu‐CDs‐BC: (d1) Bright‐field image, (d2) Fluorescence image under blue light excitation, (d3) A merged image of the images (d1) and (d2).

The pristine BC and Glu‐CDs‐BC were obtained through in situ fermentation of *K. sucrofermentans* in the conventional Hestrin‐Schramm (HS) culture medium and the conventional HS culture medium with the addition of Glu‐CDs, respectively. The photographs for the fermentation process of BC and Glu‐CDs‐BC on the days of 3, 5, 7, and 9 were shown in Figure S8 (Supporting Information). On the third day, a BC pellicle and a Glu‐CDs‐BC pellicle both have formed at the air‐culture medium interface, and their thickness was gradually increased over the time. After adding Glu‐CDs, the yield of BC decreased slightly from 6.7 mg mL^−1^ to 5.5 mg mL^−1^ (Table S1, Supporting Information). As shown in **Figure** **4**A, when observed under indoor light, the color of the purified BC pellicle was white (Figure 4A‐a1), while the color of purified Glu‐CDs‐BC pellicle appeared yellow (Figure 4A‐a2). However, when observed under UV light, pure BC did not show any fluorescence (Figure 4A‐a3), on the contrary, bright cyan emission was observed from the Glu‐CDs‐BC (Figure 4A‐a4). A few fibers obtained from the Glu‐CDs‐BC pellicle were further observed using an inverted fluorescence microscope. As shown in Figure S9 (Supporting Information), these Glu‐CDs‐BC fibers emitted green fluorescence under the excitation of blue light, while under the excitation of green light, the fibers emitted bright red fluorescence. The fluorescence spectra showed that Glu‐CDs‐BC exhibited fluorescence emission correlated with the excitation wavelength (Figure 4B). With the excitation wavelength increasing from 280 to 500 nm, the emission peak wavelength of the Glu‐CDs‐BC shifted from 515 to 590 nm, with changes in the fluorescence intensity. Specifically, the optimal excitation wavelength was ≈440 nm, and its optimal emission peak was at 572 nm.

To further confirm that the Glu‐CDs have biochemically participated in the fermentation process, *K. sucrofermentans* were incubated in the HS medium with the Glu‐CDs (1 mg mL^−1^) for 24 h. Then, they were observed under an inverted fluorescence microscope (the *K. sucrofermentans* were thoroughly washed before being dried on a glass slide). As shown in Figure 4C and *K. sucrofermentans* exhibited significant green fluorescence under the blue excitation, indicating the successful uptake of the Glu‐CDs by *K*. *sucrofermentans*. Notably, variations in fluorescence intensity were observed among *K. sucrofermentans*, indicating the ingestion of Glu‐CDs was not uniform. Subsequently, the obtained Glu‐CDs‐BC pellicle without purification was directly observed under an inverted fluorescence microscope to determine the fluorescence of *K. sucrofermentans*. As shown in Figure 4D, there was also a significant difference in fluorescence intensity among *K. sucrofermentans* in Glu‐CDs‐BC, demonstrating that these variations were not caused by purification. The *K. sucrofermentans* can uptake the Glu‐CDs and use them to produce fluorescent BC. In addition, the Glu‐CDs‐BC fibers produced by the *K. sucrofermentans* with stronger fluorescence also displayed stronger fluorescence.

The obtained Glu‐CDs‐BC was purified by treating them with a 0.2% (w/v) lysozyme solution at 30 °C for 2 h.^[^ ^13^ ^]^ Subsequently, the Glu‐CDs‐BC was thoroughly washed with Milli‐Q water until no fluorescence was detected in the residual water. To confirm the effective removal of free Glu‐CDs from the BC through the above purification process, some pristine BC was soaked and mixed with Glu‐CDs suspension for 9 days, and this sample was named Glu‐CDs/BC. This Glu‐CDs/BC sample was then purified using the above purification process. After purification, no any cyan fluorescence was observed from the Glu‐CDs/BC under UV irradiation (Figure S10, Supporting Information), and the fluorescence spectra (Figure S11, Supporting Information) and TEM analysis (Figure S12, Supporting Information) further confirmed that the purification process can effectively remove the physically attached Glu‐CDs from the BC. Thus, if any Glu‐CDs were still observable on the Glu‐CDs‐BC after the purification process, these Glu‐CDs were not attached to the BC through physical adsorption but likely formed covalent bonds with the cellulose. To further exclude the possibility of the possibility that Glu‐CDs are physically trapped within the BC network, we conducted control experiments by adding unmodified CDs to the culture medium. The resulting BC showed no fluorescence after purification, and TEM imaging revealed no surface protrusions or embedded CDs (Figure S13, Supporting Information). These results indicate that CDs lacking glucose modification are not retained within the BC network, further suggesting that they cannot be stably captured through physical interaction.

**Figure 5:**
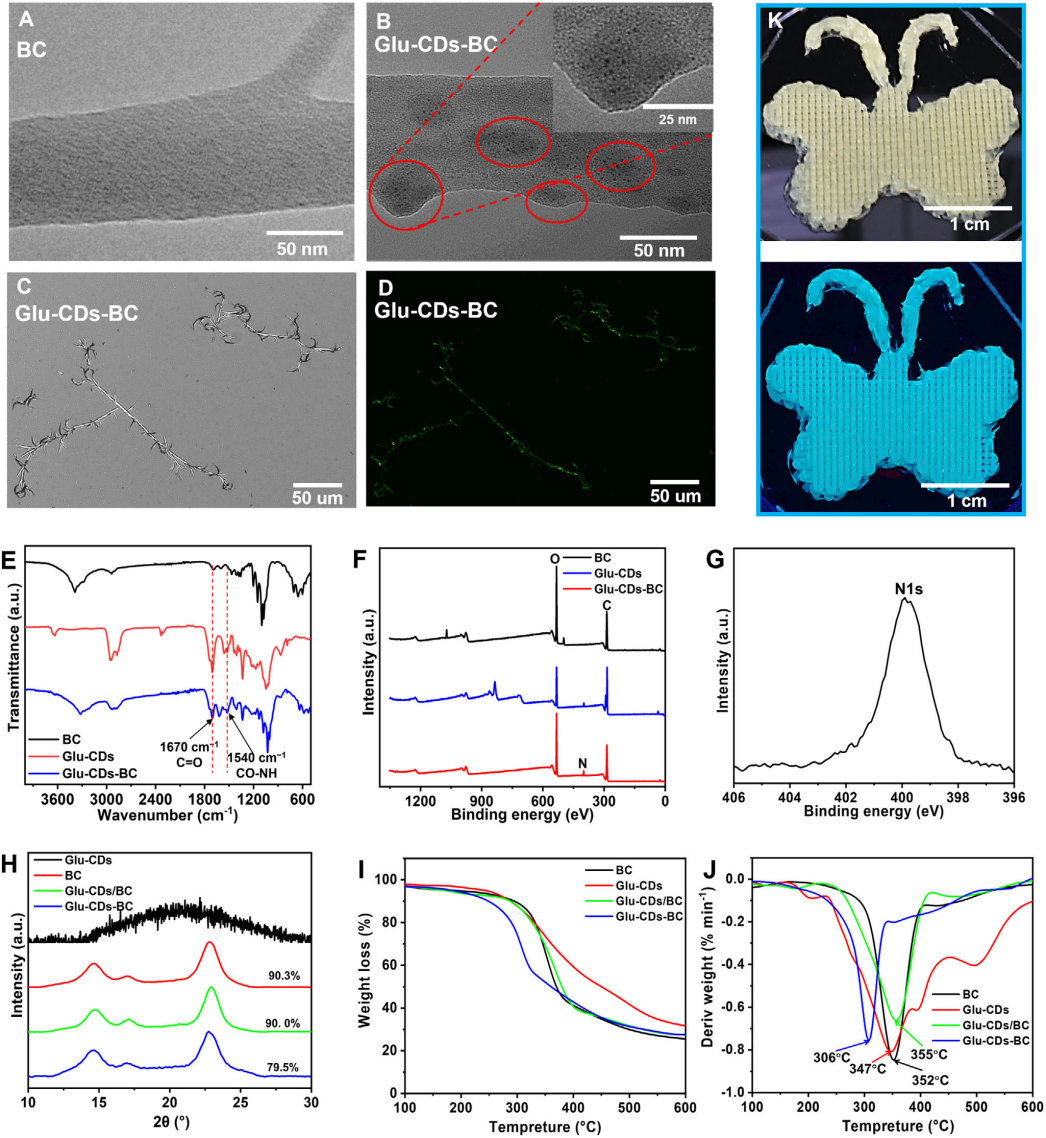
A) TEM image of the BC. B) TEM image of the Glu‐CDs‐BC. C) Bright‐field image of the Glu‐CDs‐BC by an inverted fluorescence microscope. D) Fluorescence image of the Glu‐CDs‐BC by an inverted fluorescence microscope. E) The FTIR spectra of the BC, Glu‐CDs, and Glu‐CDs‐BC. F) The XPS spectra of the BC, Glu‐CDs, and Glu‐CDs‐BC. G) The high‐resolution N1s XPS spectrum of the Glu‐CDs‐BC. H) The XRD patterns of the Glu‐CDs, BC, Glu‐CDs/BC, and Glu‐CDs‐BC. I) TGA curves of the BC, Glu‐CDs, Glu‐CDs/BC, and Glu‐CDs‐BC. J) Differential thermogravimetric (DTG) curves of the BC, Glu‐CDs, Glu‐CDs/BC, and Glu‐CDs‐BC. K) The photographs of a 3D butterfly pattern fabricated via 3D printing using Glu‐CDs‐BC‐SA hydrogel and its images under 365 nm UV light.

The SEM analysis showed that the Glu‐CDs‐BC had a 3D porous network structure (Figure S14B, Supporting Information) which is very similar to the pristine BC (Figure S14A, Supporting Information). However, the average fiber diameter of Glu‐CDs‐BC significantly increased to 41.95 ± 11.54 nm, compared to 35.32 ± 12.04 nm for pristine BC (Figures S14C–F, Supporting Information). This increase in diameter may be attributed to the incorporation of Glu‐CDs, which likely interfered with the ordered self‐assembly of cellulose chains, resulting in the formation of thicker nanofibers. In addition, the BC had a smooth surface (**Figure** **5**A), while the surface of the Glu‐CDs‐BC exhibited many convex structures. Aggregates of Glu‐CDs can be clearly observed within these protrusions (Figure 5B). Furthermore, STEM‐EDX analysis reveals the accumulation of nitrogen in these regions, supporting the localization of Glu‐CDs (Figure S15, Supporting Information). Unlike the pure glucose which is a small molecule, the nanosized Glu‐CDs may disrupt the ordered arrangement of glucose chains and result in these convex structures.^[^ ^15^ ^]^ The images of Glu‐CDs‐BC captured using an inverted fluorescence microscope also showed the unevenly distributed aggregations of Glu‐CDs formed inside of the nanofibers of the Glu‐CDs‐BC (Figures 5C,D). The above results indicate that the Glu‐CDs were not uniformly distributed in the Glu‐CDs‐BC at both the nanometer scales and micrometer scales. On the contrary, the Glu‐CDs/BC obtained by simply mixing the BC with the Glu‐CDs showed uniform fluorescence (Figure S16, Supporting Information), which can be completely eliminated after purification process (Figure S17, Supporting Information). This further indicates that the Glu‐CDs have been directly participated in the BC biosynthesis process in the Glu‐CDs‐BC samples, rather than simply being physically attached to the surface of BC.

Compared to the pristine BC, the FTIR spectra showed new peaks at 1670 and 1540 cm^−1^ formed for the Glu‐CDs‐BC (Figure 5E), which were attributed to the C═O and C─N in the amide bonds.^[^ ^11^, ^15^, ^16^, ^17^ ^]^ Since the purification process can effectively remove the Glu‐CDs which were physically attached to the BC, the presence of amide bonds in the Glu‐CDs‐BC indicates that Glu‐CDs have been incorporated to the Glu‐CDs‐BC through covalent bonds rather than adsorption. The chemical compositions of the BC and Glu‐CDs‐BC were analyzed by XPS. As shown in Figure 5F, the BC comprised of C (62.45%) and O (37.55%), while the Glu‐CDs‐BC encompassed C (63.55%), O (35.05%) and N (0.4%). The high‐resolution N spectrum of the Glu‐CDs‐BC displayed a single peak at 399 eV (Figure 5G), corresponding to the C─N signal peak,^[^ ^39^ ^]^ aligns with the FTIR results, confirming the presence of the amide bond and successful incorporation of the Glu‐CDs into the Glu‐CDs‐BC. The Brunauer‐Emmett‐Teller (BET) analysis shows that the incorporation of Glu‐CDs led to a slight increase in the average pore diameter of Glu‐CDs‐BC compared to pristine BC, which in turn resulted in a decrease in specific surface area (Figure S18, Supporting Information). The XRD analysis showed that the BC, Glu‐CDs/BC, and Glu‐CDs‐BC all had two peaks at 14.5° and 22.6°, which were corresponded to the (110) and (200) faces of cellulose I‐β crystals,^[^ ^40^, ^41^ ^]^ respectively (Figure 5H). The XRD results indicated that the incorporation of the Glu‐CDs had no significant effect on the crystal structure type of the BC. However, the crystallinity of Glu‐CDs‐BC (79.5%) was much lower than the pristine BC (90.3%). The decrease in crystallinity of Glu‐CDs‐BC indicated the formation of less crystalline regions or more amorphous regions, as it is consistent with the convex structures observed on the Glu‐CDs‐BC in the TEM images (Figure 5B), indicating that incorporation of Glu‐CD during the Glu‐CDs‐BC biosynthesis disrupts the ordered arrangement of the cellulose chains, resulting in the formation of more amorphous regions and thus leading to a decrease in crystallinity. Additionally, the crystallinity of the Glu‐CDs/BC was 90%, which was very close to the pristine BC (90.3%). However, the content of Glu‐CDs in Glu‐CDs/BC is 6.82 wt%, which is much higher than that in Glu‐CDs‐BC (0.61 wt.%) (the calculation of the contents of Glu‐CDs in the Glu‐CDs/BC and the Glu‐CDs‐BC was described in Figure S19 (Supporting Information). These results indicate that the simple physical adsorption of Glu‐CDs, even up to 6.82 wt%, on the BC surface did not have a significant effect on the crystallinity of the BC. Therefore, the crystallinity of Glu‐CDs‐BC would not decrease even if all these 0.61 wt% of Glu‐CDs were physically adsorbed on the BC. This further confirms that the decrease in crystallinity of the Glu‐CDs‐BC was not caused by the Glu‐CDs physically adsorbed on the surface of Glu‐CDs‐BC. It is worth noting that excessive addition of Glu‐CD in the culture concentration may increase the loading of Glu‐CDs‐BC, but it will greatly reduce its yield (Table S2, Supporting Information).

To further confirm the Glu‐CDs were successfully engaged in the biosynthesis of cellulose, other than physical adsorption on the surface of BC, TGA was performed on the Glu‐CDs/BC and Glu‐CDs‐BC. As shown in Figure 5I, the TGA results indicated that the initial thermal decomposition temperature for the BC was 293 °C, for the Glu‐CDs was 298 °C, and for the Glu‐CDs/BC was 290 °C, but for the Glu‐CDs‐BC was only 215 °C. The derivative thermal decomposition temperatures showed that the temperature at the maximum weight loss rate for the BC was 352 °C, for the Glu‐CDs was 347 °C, and for the Glu‐CDs/BC was 355 °C, but for the Glu‐CDs‐BC was only 306 °C (Figure 5J). The above thermal analysis results showed that the initial decomposition temperature and the temperature at the maximum weight loss rate for the BC and the Glu‐CDs/BC were very close, indicating that by simply absorbing of the Glu‐CDs on the BC would not change the thermal stability of the BC. However, both of the initial decomposition temperature and the temperature at the maximum weight loss rate for the Glu‐CDs‐BC were much lower than that of the BC and the Glu‐CDs/BC, indicating that the Glu‐CDs were not physically absorbed on the Glu‐CDs‐BC, but biochemically participated in the biosynthesis process, leading to the formation of more amorphous regions of the Glu‐CDs‐BC, and consequently reducing the thermal stability of the Glu‐CDs‐BC.^[^ ^15^ ^]^ The TGA results was consistent with the TEM and XRD analyses, providing additional evidence that glucose‐modified CDs nanoparticles can be biochemically engaged with and utilized by microorganisms.

### 2.5 Glu‐CDs‐BC Used for 3D Printing of 3D Fluorescent Structures

The 3D printing of fluorescent materials has potential applications in various fields such as anti‐counterfeiting, sensing, and medicine. To demonstrate the capabilities of Glu‐CDs‐BC in constructing complex 3D structures, we prepared a printable composite hydrogel (Glu‐CDs‐BC‐SA) by simply mixing Glu‐CDs‐BC with sodium alginate (3% in dry weight). The Glu‐CDs‐BC‐SA exhibited excellent shear‐thinning behavior, characterized by a decrease in viscosity with increasing shear rate (Figure S20, Supporting Information). The Glu‐CDs‐BC‐SA hydrogel enabled smooth extrusion through a 3D printer while maintaining robust structural integrity without collapse. As shown in Figure S21A (Supporting Information), the 3D square frustum structure printed with Glu‐CDs‐BC‐SA hydrogel had a high fill rate (70%) and retained a stable shape. Additionally, Glu‐CDs‐BC‐SA hydrogel can also print low fill rate (20%), hollow cuboid structures (Figure S21B, Supporting Information) that maintained their shape without visible deformation. The Glu‐CDs‐BC‐SA hydrogel is suitable not only for regular geometric shapes but also for complex patterns, such as the 3D butterfly pattern (Figure 5K). Due to the intrinsic fluorescence of Glu‐CDs‐BC, the 3D structures printed with the Glu‐CDs‐BC‐SA hydrogel exhibited cyan fluorescence under UV light (Figure 5K). Compared to the physical adsorption of fluorescent molecules, since the CDs are covalently embedded within the BC and isolated from the surrounding environments, the fluorescent properties of these 3D structures are more stable and will not diffuse into the surrounding solvent environment, thus, it is especially favorable for their use in biomedical applications, which can effectively minimize the potential impacts on biological systems.

We have developed an innovative method for the in situ biosynthesis of fluorescent cellulose with glucose‐modified CDs nanoparticles using only microorganisms. These Glu‐CDs are covalently linked to cellulose chains through cellulase enzymes within the microorganisms, shining the cellulose with a unique cyan fluorescence. In contrast to conventional physical and chemical modification techniques, our biosynthetic strategy offers several advantages.^[^ ^11^, ^15^ ^]^ First, it is environmentally friendly. Unlike chemical grafting, which often involves high temperatures, strong acids or bases, or toxic organic solvents, our method was performed under mild conditions (room temperature and atmospheric pressure) without the need of hazardous reagents. Second, the incorporation of Glu‐CDs is achieved through covalent bonding during the in situ biosynthesis of BC. This covalent incorporation ensures stronger binding compared to physical adsorption, which relies on weak intermolecular interactions and may result in nanoparticle detachment over time, leading to deteriorated material performance. Furthermore, although the Glu‐CDs content in Glu‐CDs‐BC is relatively low (0.61 wt%), the Glu‐CDs are incorporated inside the cellulose fibrils by covalent integration along the glucose chains. This is in contrast to chemical or physical methods, which typically graft or adsorb CDs only on the surface. Our method thus preserves the surface accessible of BC for other modifications. Interestingly, the Glu‐CDs exhibit a non‐uniform distribution within the nanofibers, which may provide unique advantages in applications such as information encryption, where spatial heterogeneity can be beneficial. Our approach not only enables the environmentally friendly production of sustainable and functional materials based on biosynthesis but also established the potential for using nanoparticles as building blocks in microorganisms, rather than relying on small molecules.

## 3 Conclusion

In summary, this study demonstrates the effective incorporation of nanoparticles into bacterial cellulose (BC) through microbial fermentation. By introducing glucose‐modified CDs (Glu‐CDs) as a nutrient source in the culture medium, we achieved the in situ biosynthesis of a cyan fluorescent carbon dot‐bacterial cellulose composite (Glu‐CDs‐BC). Notably, Glu‐CDs are covalently bonded to cellulose chains via cellulase enzymes within the microorganisms, distinguishing this method from traditional approaches that rely on physical adsorption or chemical reactions. To our knowledge, this is the first successful incorporation of functional nanoparticles, specifically CDs, through cellulase‐catalyzed processes in microbial cell factories of BC. Multiple evidences including microscopic imaging, structural analysis, and control experiments, support the hypothesis that Glu‐CDs are covalently integrated into the cellulose chains during biosynthesis, rather than being physically adsorbed or entangled. This approach not only enhances the sustainable modifications of cellulose‐based composites but also introduces new methodologies and perspectives for the broader fields of biosynthesis. Furthermore, Glu‐CDs‐BC can be simply converted into a printable hydrogel with sodium alginate, and various 3D fluorescent structures were successfully fabricated with this composite hydrogel through 3D printing. This capability indicates its promising potential for a new generation of sustainable and advanced materials.

## 4 Experimental Section

### Materials

Unless otherwise noted, all reagents were used without purification. citric acid (AR 99.7%), glucosamine, 4‐(4, 6‐Dimethoxy‐1, 3, 5‐triazin‐2‐yl)‐4‐methylmorpholin‐4‐ium chloride (DMTMM, AR 99.7%), sodium alginate (SA, 15–60 mPa⋅s, Mw is 20000–50000 Da), and methanol (AR 99.7%) were purchased from Shanghai Macklin Chemical Co., Ltd. All chemicals were used as received. Milli‐Q water was used in all experiments. *K*. *sucrofermentans* was obtained from BeNa Culture Collection. Hestrin‐Schramm (HS) culture medium purchased from Shandong Tuopu Bio‐engineering Co., Ltd.

### Preparation of CDs

Citric acid (5 g) was added into a round‐bottomed flask and heated at 180 °C for 48 h. The obtained solid powder was washed three times with Milli‐Q water, then was dispersed in 500 mL of Milli‐Q water and centrifuged at 8000 rpm for 5 min, then the supernatant was collected and freeze‐dried to obtain a yellow powder.

### Preparation of Glu‐CDs

The obtained CDs (250 mg), glucosamine (250 mg), and DMTMM (500 mg) were added to methanol (5 mL), stirred at room temperature for 6 h, and then dialyzed for 2 days using a dialysis bag with a molecular weight cutoff of 1000 Da. The dialyzed liquid was freeze‐dried to obtain Glu‐CDs.

### Biosynthesis of BC

To prepare the culture medium, 38.9 g of HS medium was mixed with 1L of deionized water, and then the solution was placed in an autoclave (Zhiwei A, Xiamen, China) at 121 °C for 20 min. *K. sucrofermentans* was cultured with high‐pressure and high‐temperature sterilized HS basic medium at 30 °C for 9 days. Then, the obtained BC was treated with lysozyme solution (0.2%, w/v, 5 mL) at 30 °C for 2 h and then washed thoroughly with Milli‐Q water.

### Biosynthesis of Glu‐CDs‐BC Using Only Microorganisms

The *K. sucrofermentans* was cultured with high‐pressure and high‐temperature sterilized HS basic medium supplemented with Glu‐CDs (1 mg mL^−1^) at 30 °C for 9 days. Then, the obtained Glu‐CDs‐BC was treated with lysozyme solution (0.2%, w/v, 5 mL) at 30 °C for 2 h and then washed with Milli‐Q water thoroughly until no fluorescence was detected in the residual water.

### Preparation of CDs/BC

The *K. sucrofermentans* was cultured with high‐pressure and high‐temperature sterilized HS basic medium supplemented with CDs (1 mg mL^−1^) at 30 °C for 9 days. Then, the obtained CDs/BC was treated with lysozyme solution (0.2%, w/v, 5 mL) at 30 °C for 2 h and then washed with Milli‐Q water thoroughly until no fluorescence was detected in the residual water.

### Preparation of Glu‐CDs/BC

The Glu‐CDs/BC was prepared by immersing raw BC in Glu‐CDs suspension (1 mg mL^−1^) at 30 °C for 9 days followed by filtration, without any further washing.

### Inkjet 3D Printing of Glu‐CDs‐BC‐SA

To prepare a printable hydrogel ink, the Glu‐CDs‐BC suspension was first mechanically disintegrated using a high‐speed blender for 15 min. The resulting mixture was then concentrated in a drying oven to obtain a 10 wt.% suspension. Subsequently, 0.5 g of sodium alginate powder was added into 10 g of a 10 wt% Glu‐CDs‐BC aqueous suspension and vigorously stirred to form a homogeneous and viscous mixture. Water was used as the only solvent in this process. The direct ink writing (DIW) process was conducted using a System 30 m 3D printer (Axolotl Biosystems) equipped with a reservoir‐based print head (5 mL syringe). For all printing experiments, an 18‐gauge blunt‐tip needle (ID = 0.84 mm, length = 1.5 in) was used. The square frustum and hollow cuboid structures were designed using AutoCAD, while the butterfly pattern and corresponding STL files were obtained from Thingiverse. Optimal printing parameters were applied as follows: printing speed of 10 mm s^−1^, layer height of 0.4 mm, and a 0.4 mm gap between the needle tip and printing surface.

### Characterization Methods

The morphology of the BC and Glu‐CDs‐BC were investigated by SEM (SU8010, Hitachi Ltd., Japan), the samples were mounted onto aluminum stubs, coated with a layer of platinum by Ion sputtering instrument (MC1000, Hitachi Ltd., Japan), and analyzed using SEM with a 3.0 kV acceleration voltage. The CDs, BC and Glu‐CDs‐BC were also observed by TEM (HT‐7700 Exalens, Hitachi Ltd., Japan), to prepare the specimens, the CDs were dispersed in Milli‐Q water with a concentration of 10 µg mL^−1^, then 10 µL of the CDs suspension was dropped onto the surface of an ultra‐thin carbon coated copper grid and dried completely, the BC and Glu‐CDs‐BC were dispersed in Milli‐Q water with a concentration of 1 µg mL^−1^, then 10 µL of the BC or Glu‐CDs‐BC suspension was dropped onto copper grids with lacey support films and dried completely. Dimensions of CDs was measured by TEM and analyzed using ImageJ. The fluorescence spectra of the CDs, Glu‐CDs and Glu‐CDs‐BC were obtained using a fluorescence spectrophotometer (FS5, Edinburgh Ltd., UK). The absorption spectra of the CDs and Glu‐CDs were obtained by UV–Vis‐NIR spectrophotometer (UV‐3600 plus, Shimadzu Ltd., Japan). The crystallinity of the CDs, Glu‐CDs, BC, and Glu‐CDs‐BC were measured by XRD (D8 Advance, Bruker AXS., Germany). Fourier transform infrared (FTIR) spectra were obtained on a Nicolet 6 VERTEX 70 FTIR spectrometer (Bruker AXS., Germany). X‐ray photoelectron spectroscopy (XPS) was performed on a K‐Alpha electron spectrometer (Thermo Fisher Scientific Ltd., USA). The Raman spectra of the CDs were recorded in Via spectrometer (Renishaw Via) with an excitation wavelength of 532 nm. The TGA measurements were performed on a TGDTA7300 thermal analyzer by heating under a flow of N_2_ gas from room temperature to 900 °C at a rate of 10 °C min^−1^. Thermal behaviors were also characterized by differential scanning calorimetry (DSC, Q2000, TA Instruments, United States) at a ramp rate of 5 °C min^−1^. The Zeta potential and hydrodynamic diameter of the CDs were obtained using a Zeta size analyzer (Nano ZS zen3600, Malvern Instruments Ltd.). Fluorescence images were taken using a Leica DMi8 inverted fluorescence microscope (Leica, Germany). Rheological measurements (TA Instruments, Discovery HR‐2) were conducted using a flat and plate geometry (20 mm, 2° plate) at 25 °C. Flow tests were carried out at shear rates from 0.01 s^−1^ to 1000 s^−1^. The N_2_ physisorption isotherms at 77 K were operated on Quantachrome Instruments version 3.01. Pore size distribution was calculated by the Barret‐Joyner‐Halenda (BJH) model. Specific surface area (SBET) was calculated by using Brunauer‐Emmett‐Teller (BET) equation. For STEM‐EDX sample preparation, Glu‐CDs‐BC was dispersed in Milli‐Q water with a concentration of 1 µg mL^−1^, then 10 µL of the Glu‐CDs‐BC suspension was dropped onto copper grids with lacey support films and dried completely. Morphology and local elemental distribution of Glu‐CDs‐BC were analyzed using a STEM‐EDX system (Bruker Super‐X SDDs, Germany) operated at an accelerating voltage of 200 kV, with a probe current of 0.5 nA, a convergence semi‐angle of 21 mrad, and a high‐angle annular dark‐field (HAADF) inner collection angle of 50 mrad. EDX spectrum images were acquired using Bruker Esprit software in combination with a Titan Super‐X detector system.

## Conflict of Interest

The authors declare no competing interests.

## Supporting information



Supporting Information

## Data Availability

The data that support the findings of this study are available from the corresponding author upon reasonable request.
