# Supplementary material for: Nanoparticle‐Functionalized Cellulose Through Biosynthesis‐Only Approach
Source: Adv Sci (Weinh). 2025 Sep 12;12(45):e11965. doi: 10.1002/advs.202511965 (PMC12677631; doi:10.1002/advs.202511965)
Supplement: Supplementary file 1 — Supporting Information [file ADVS-12-e11965-s001.docx]

**Supporting Information**

**Nanoparticle-Functionalized Cellulose through Biosynthesis-only Approach**

*Chunyu Ji, Ting Wang, Yifeng Wang, Qian Ding, Han Yang**

*Chunyu Ji, Ting Wang, Yifeng Wang, Qian Ding*

School of Chemical Sciences, University of Chinese Academy of Sciences,

Beijing, 100049 P. R. China.

School of Chemical Engineering, University of Chinese Academy of Sciences, Beijing, 100049 P. R. China.

*Han Yang*

School of Chemical Engineering, University of Chinese Academy of Sciences, Beijing, 100049 P. R. China.

Email: [yanghan@ucas.ac.cn](mailto:yanghan@ucas.ac.cn)


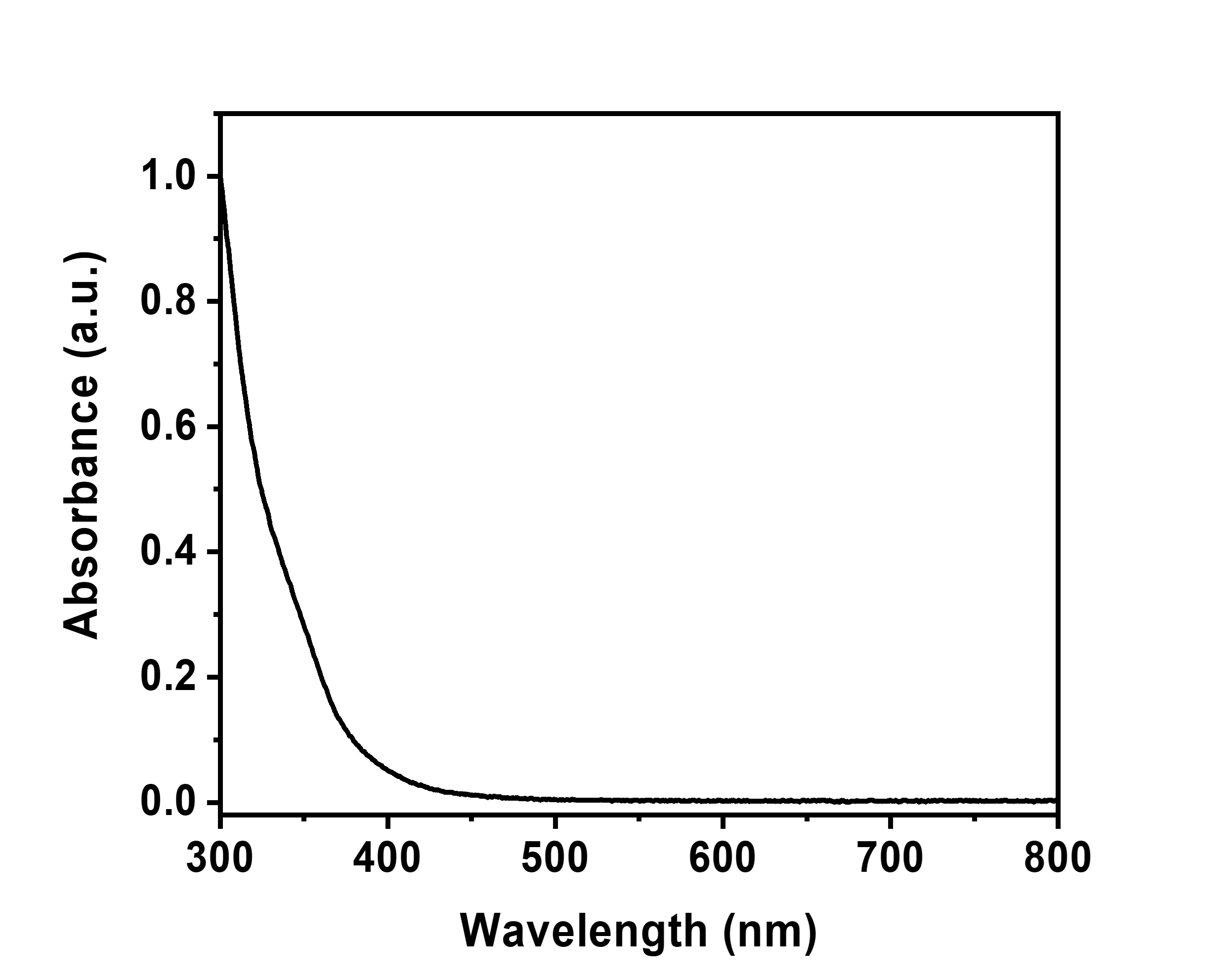


**Figure S1**. The UV-vis spectrum of the CDs.

The optical properties of the CDs were investigated by UV-vis spectrophotometer (**Figure S1**). The UV-vis spectrum showed that these CDs have a broad absorption in the UV region with a tail in the visible region, which was attributed to π–π* (C=C) and n–π* (C=O) transitions^1^.


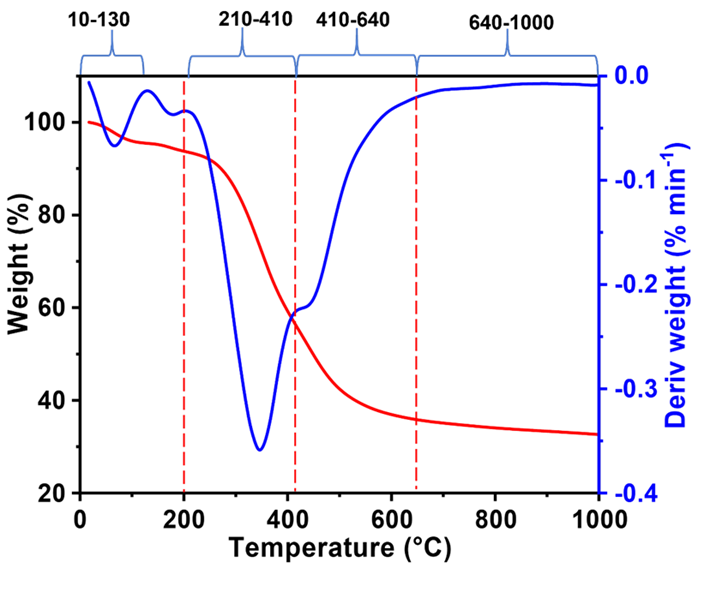


Figure S2. Thermo-gravimetric analysis curves of the CDs.

The thermal characteristics of the CDs were studied using thermo-gravimetric analysis (TGA). Four distinct regions were observed on the weight loss curve for CDs (**Figure S2**): an initial weight loss of 3.8% occurred in the temperature range of 10 - 130°C due to the evaporation of free water, followed by a small weight loss of 3% in the temperature range of 130 - 210°C due to the evaporation of bound water. Subsequently, a significant weight loss of 37.2% was observed in the temperature range of 210 - 410°C due to the oxidation of functional molecules on the surface of CDs, and a final weight loss of 21% occurred in the temperature range of 410 - 640°C due to the continued carbonization of the previously uncarbonized portion of CDs. Consequently, the residual mass of the CDs was 32.7% of the original mass.


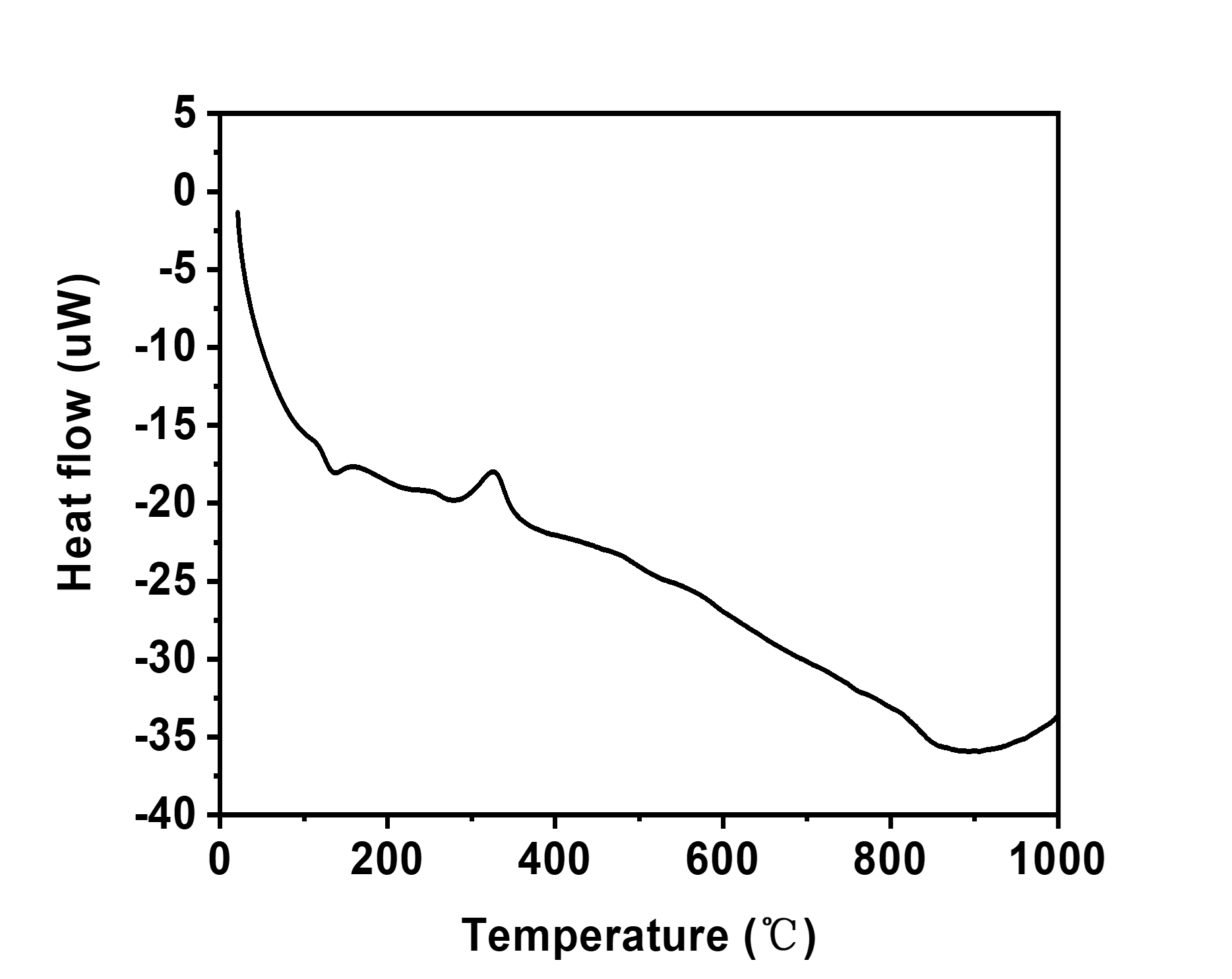


Figure S3. Differential scanning calorimetry (DSC) curve of the CDs.

The differential scanning calorimetry (DSC) curve of the CDs was measured and shown in **Figure S3**. An endothermic process observed between 10 - 210°C was primarily caused by the evaporation of free water and bound water. The subsequent exothermic process occurring within the temperature range of 210°C to 410°C was mainly attributed to the oxidation of functional molecules on the surface of the CDs. The endothermic process occurring between 410°C and 600°C was attributed to the continued carbonization of the previously uncarbonized portion of the CDs. The thermal degradation process indicated the presence of abundant non-carbonized functional components on the CDs, which tend to decomposition at elevated temperatures.


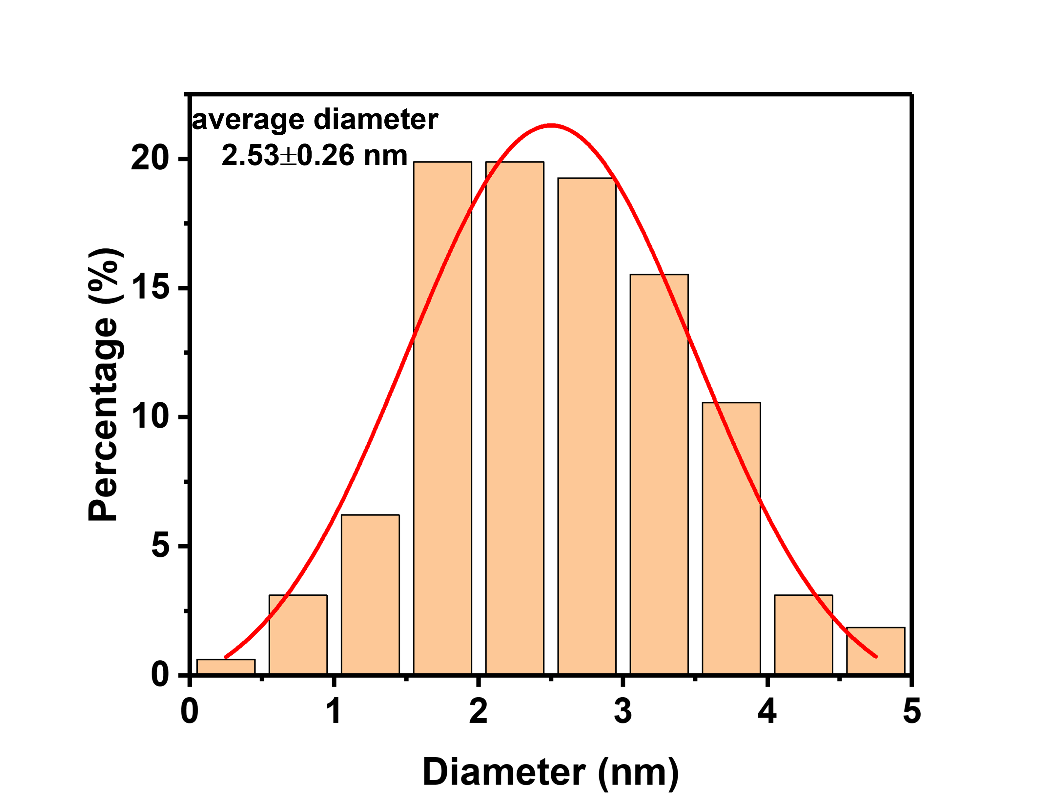


**Figure S4.** The diameter distribution histogram of the CDs.

We measured the diameter of the CDs based on the TEM images and plotted them as a histogram (**Figure S4**). The statistical results show that the diameter distribution of the CDs ranges from 0.43 nm to 4.76 nm, with an average diameter of 2.53±0.26 nm. The wide range of distribution in the size of CDs is the main reason for the variation in their fluorescence emission with excitation.


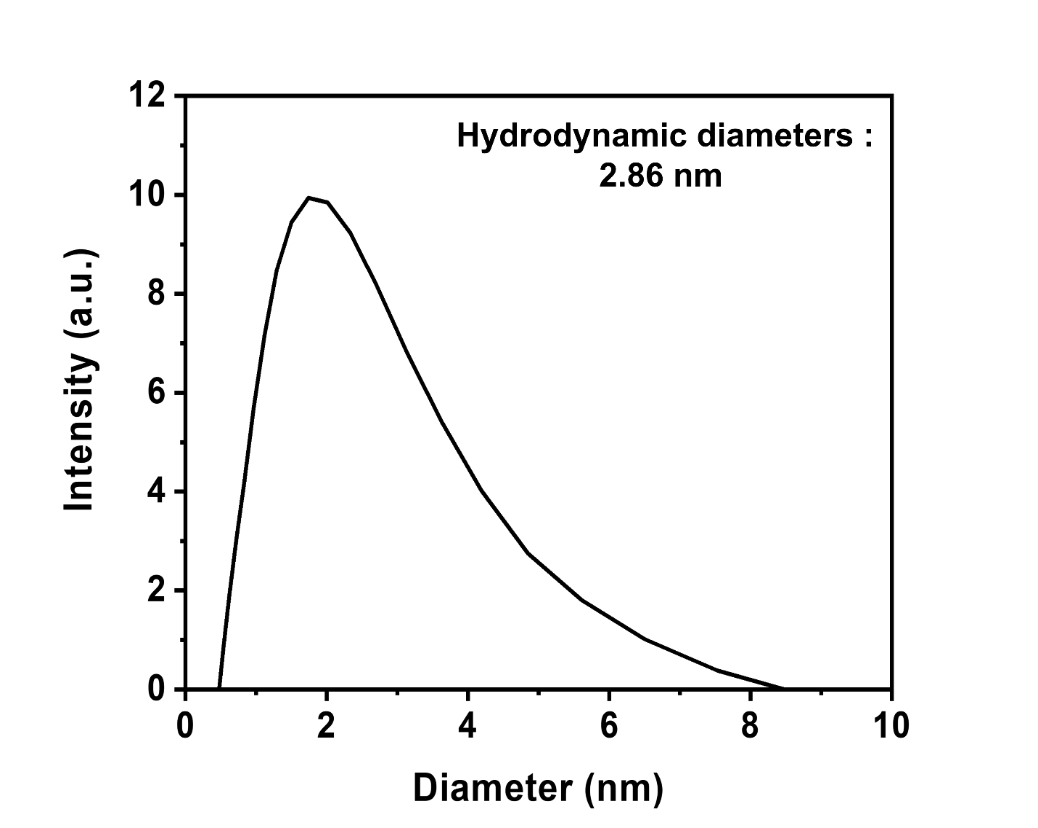


**Figure S5.** The hydrodynamic diameter distribution of the CDs.

The hydrodynamic diameter distribution of the CDs was measured using a Zetasizer Nano (Malvern, UK) utilizing dynamic light scattering (DLS). The result showed that the average hydrodynamic diameter was 2.86 nm (**Figure S5**), which was slightly greater than the diameter obtained based on the TEM analysis. This is possibly due to the non-carbonized part on the CDs can stretch in aqueous environment during the DLS measurements.


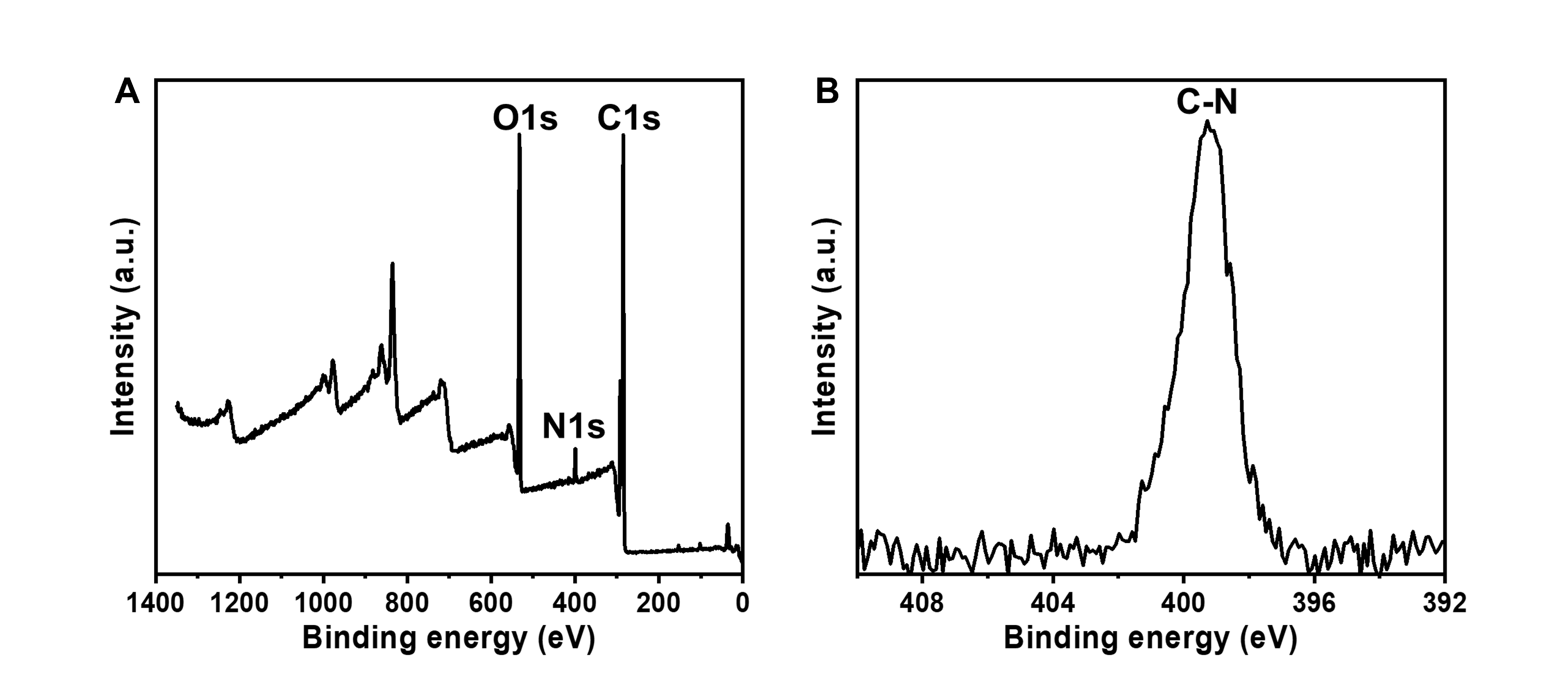


**Figure S6.** (A) The XPS survey spectrum of the Glu-CDs. (B) The high-resolution C1s XPS spectrum of the Glu-CDs.


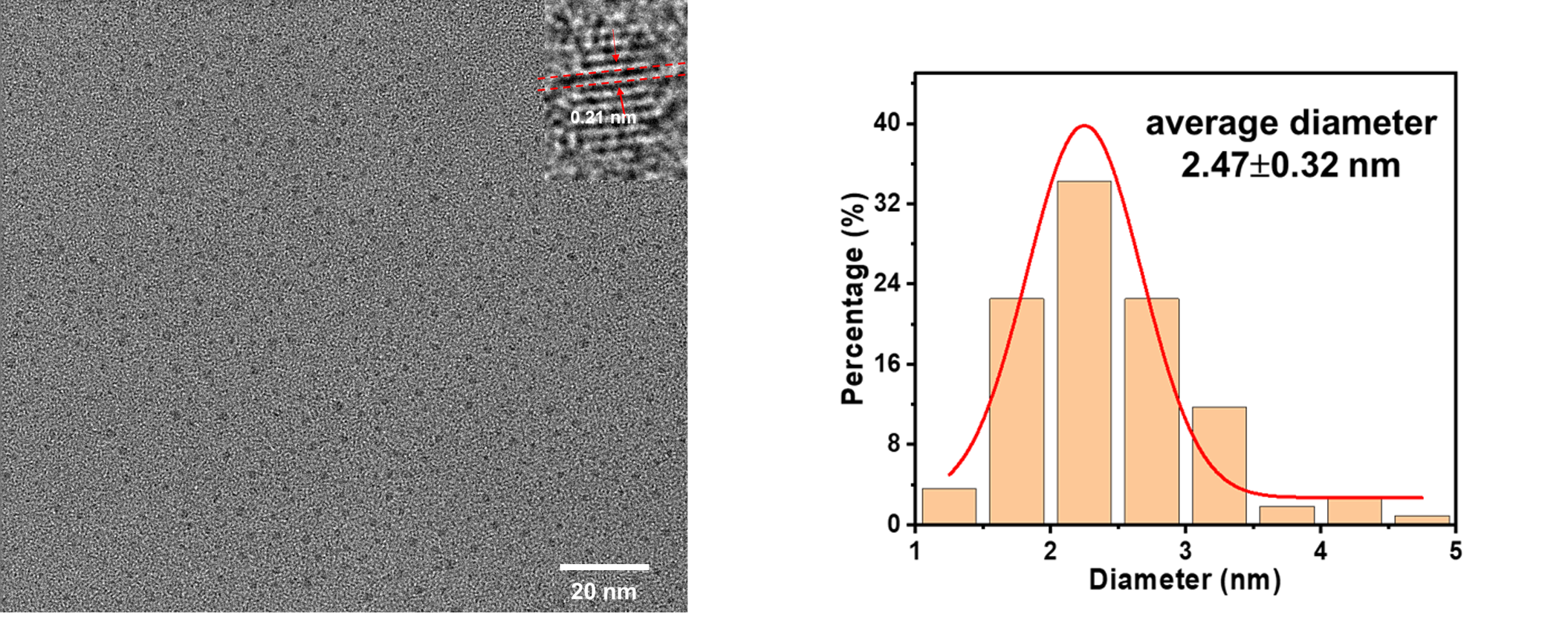


**Figure S7.** TEM image and diameter distribution histogram of the dispersed Glu-CDs.


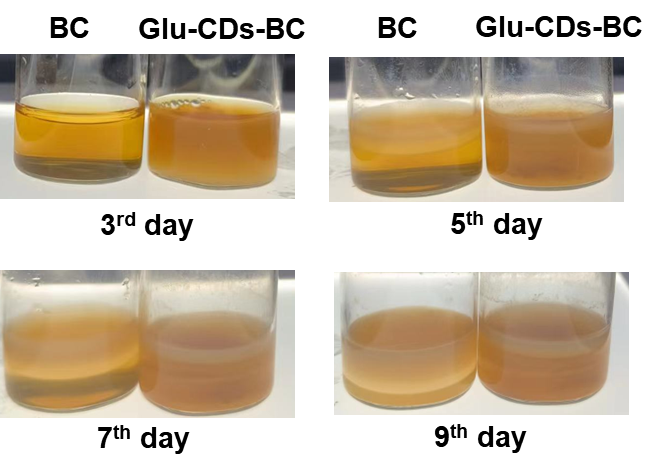


**Figure S8.** Photographs of BC and Glu-CDs-BC fermentation process under standard conditions (30°C) on days 3, 5, 7, and 9 (the left bottle is for BC, and the right bottle is for Glu-CDs-BC).


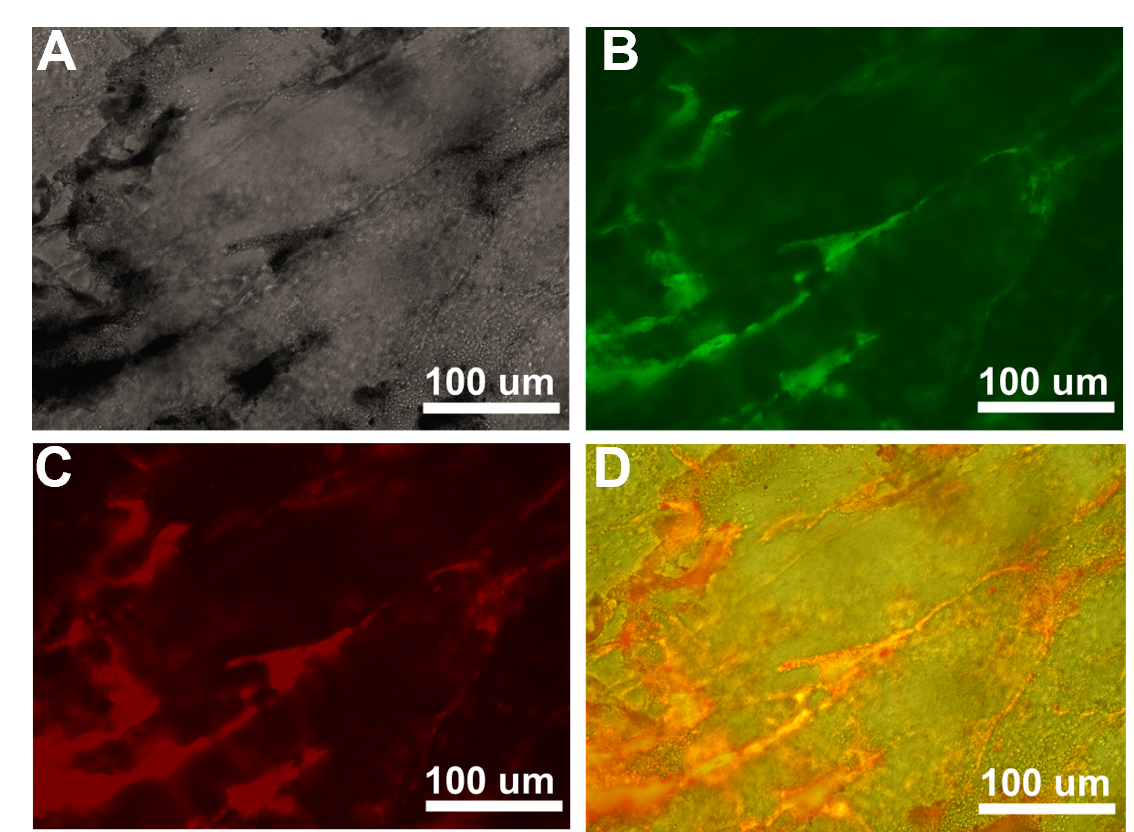


**Figure S9.** Images of fluorescence emission of Glu-CDs-BC after purification using an inverted fluorescence microscope: (A) Bright-field image of Glu-CDs-BC after purification, (B) Fluorescence image of Glu-CDs-BC under blue light excitation, (C) Fluorescence image of Glu-CDs-BC under green light excitation, (D) A merged image of the images (A), (B), and (C).


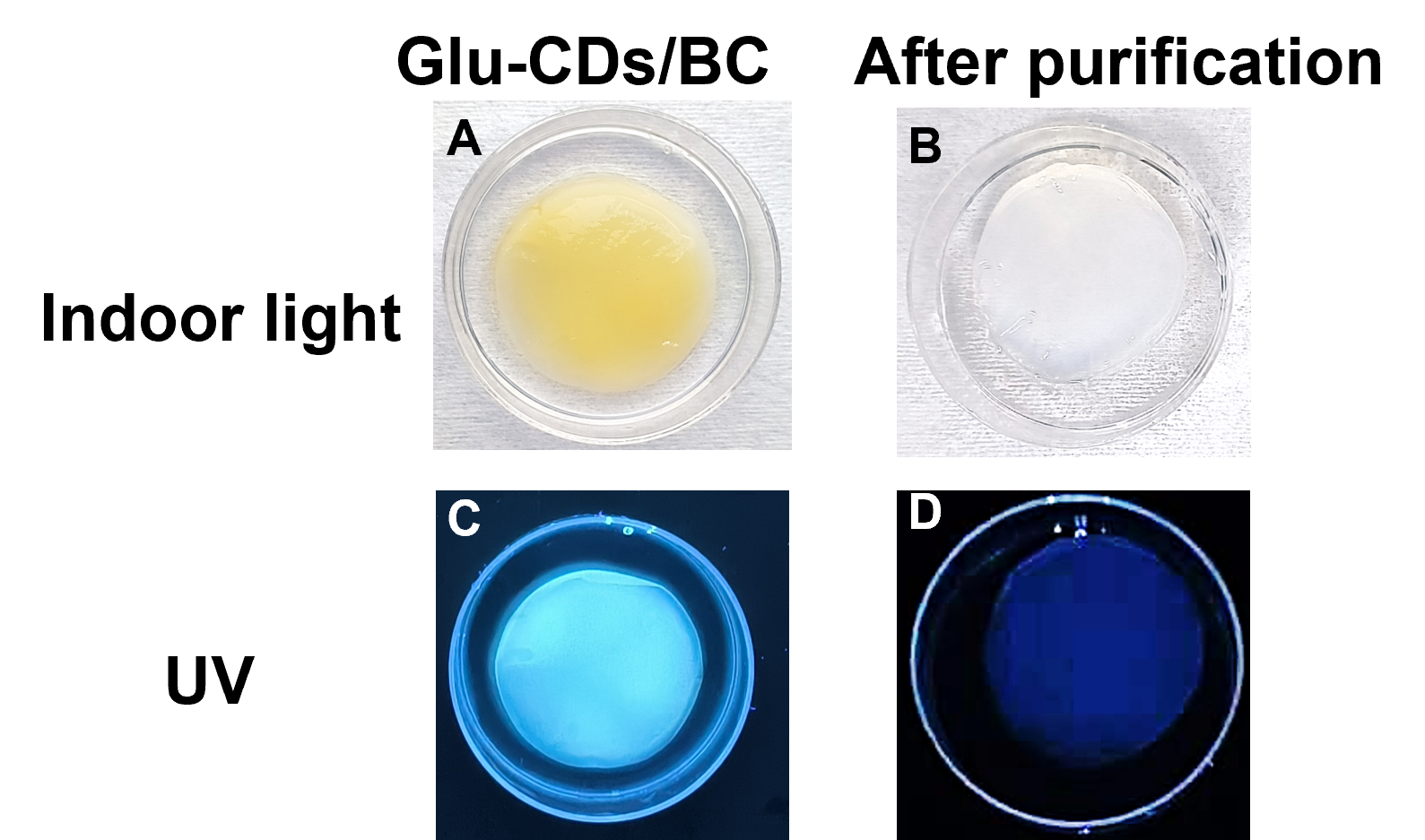


**Figure S10** Photographs of Glu-CDs/BC (A) before and (B) after purification, taken under indoor light. Photographs of Glu-CDs/BC (C) before and (D) after purification, taken under 365 nm UV light illumination.


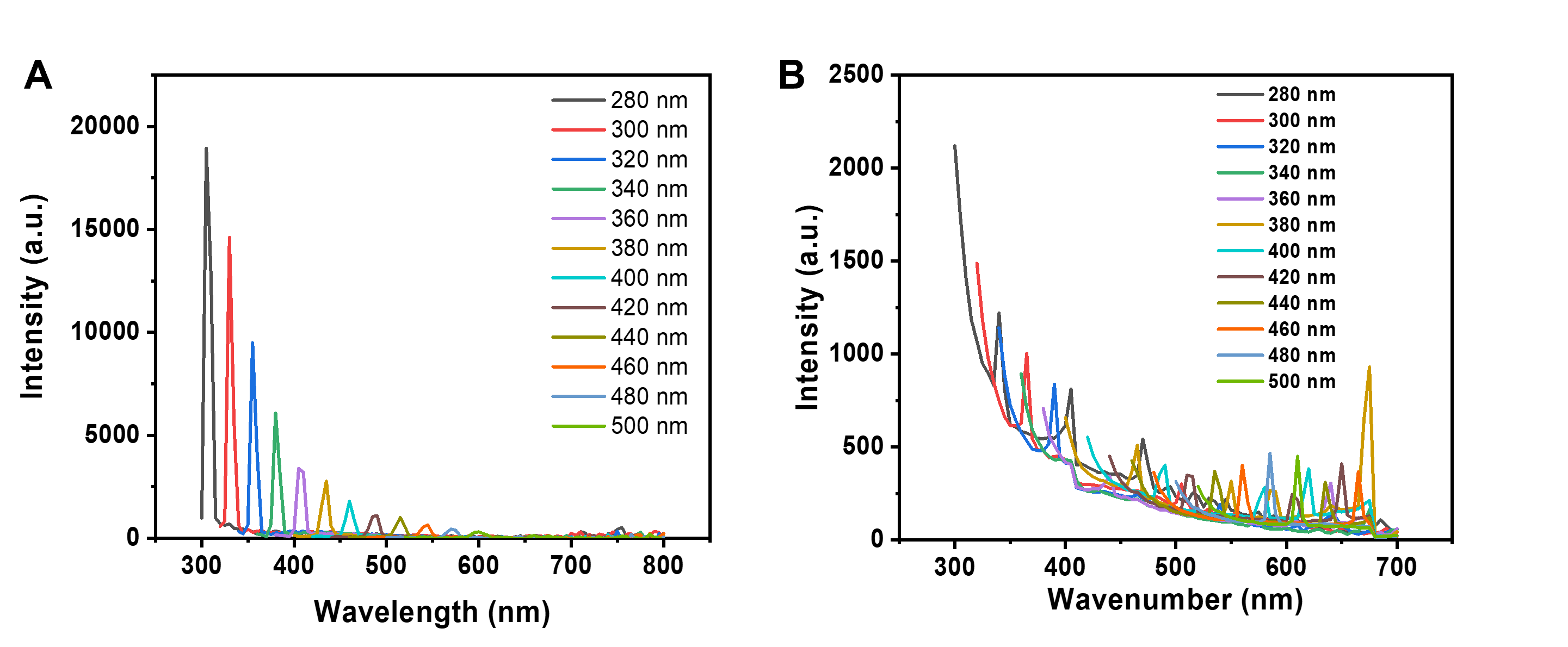


**Figure S11**. (A)Fluorescence spectra of Glu-CDs/BC after purification. (B) Fluorescence spectra of BC.


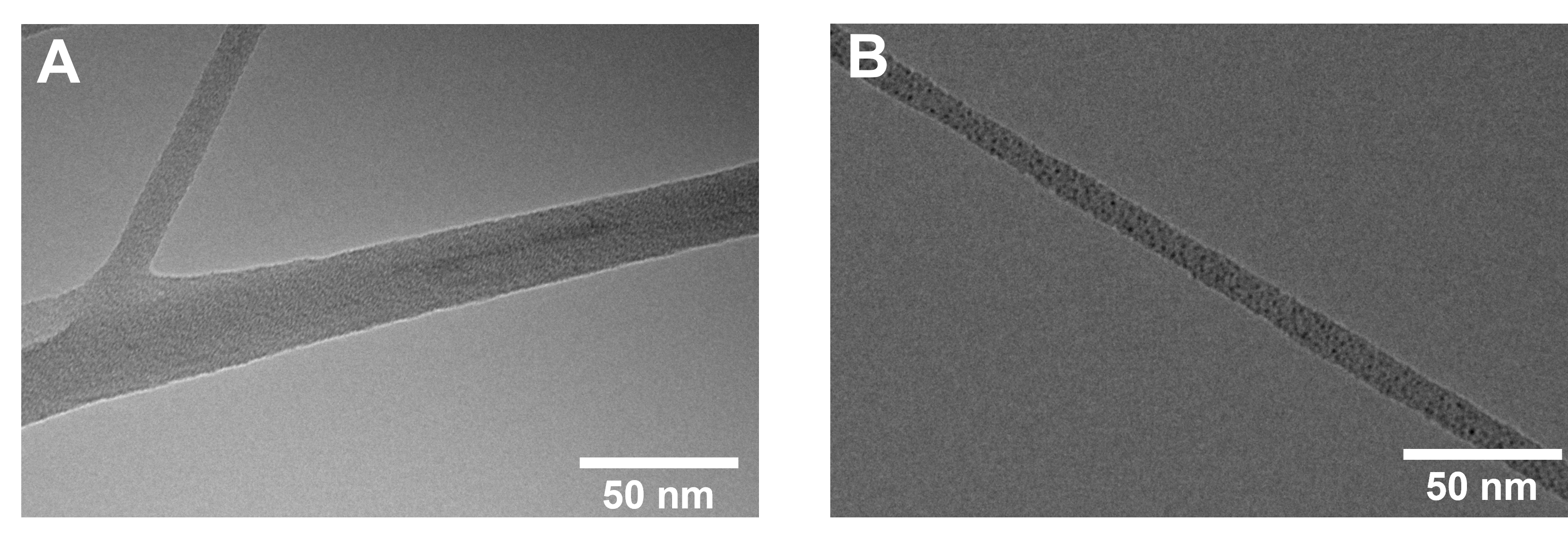


**Figure S12.** The TEM images of Glu-CDs/BC (A) after and (B) before purification.

The BC was soaked in a suspension of Glu-CDs (1 mg mL^−1^) for 9 days, and this resulting sample was named Glu-CDs/BC, which was light yellow color under indoor light (**Figure S10A**), and showed strong cyan fluorescence under 365 nm UV light illumination (**Figure S10C**). The obtained Glu-CDs/BC was purified following the same purification procedure as that used for Glu-CDs-BC. However, after purification, Glu-CDs/BC turned colorless (**Figure S10B**) and did not show fluorescence under UV light illumination (**Figure S10D**). The fluorescence spectra did not show any fluorescent characteristic peaks for purified Glu-CDs/BC (**Figure S11**). The TEM characterizations also demonstrated that dense Glu-CDs were distributed on the surface of BC fibers before purification (**Figure S12A**), however, none of the Glu-CDs could be observed on the surface of BC after purification (**Figure S12B**). Therefore, the Glu-CDs adsorbed on the surface of BC through physical interactions can be completely removed using the purification procedure we employed. Thus, there should be no physically adsorbed CDs in the Glu-CDs-BC samples after purification.


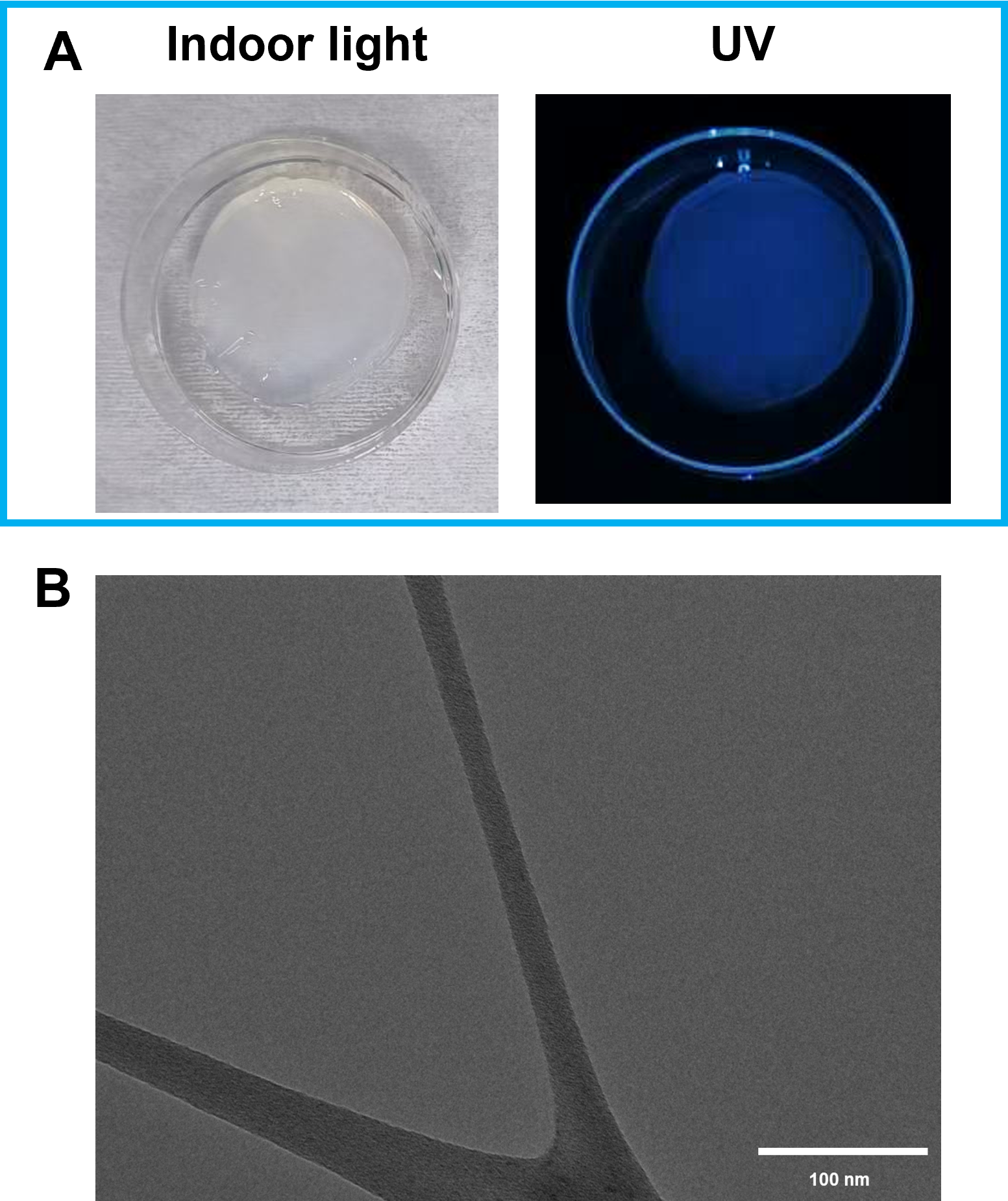


**Figure S13.** (A) Photographs of BC obtained from a CD-containing culture medium under indoor light and 365 nm UV light. (B) TEM image of the BC produced in the CD-containing culture medium after purification.


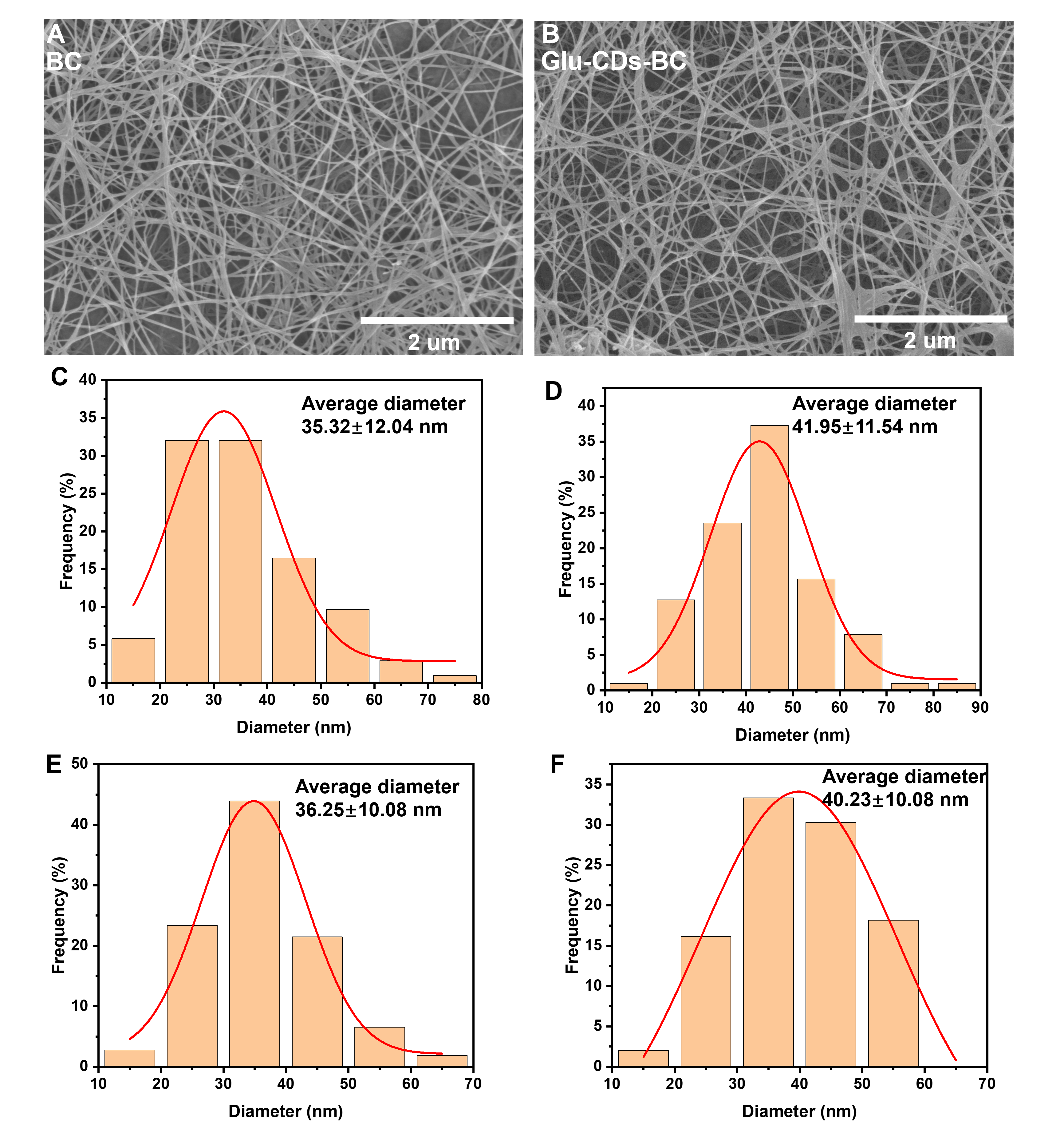


**Figure S14.** SEM image of the (A) BC and (B) Glu-CDs-BC. The diameter distribution histogram of the (C) BC and (D) Glu-CDs-BC based on SEM investigation. The diameter distribution histogram of the (E) BC and (F) Glu-CDs-BC based on the TEM investigation.

SEM analysis indicates that Glu CDs BC maintains a three-dimensional network structure analogous to pure BC, demonstrating that carbon dot modification preserves its original microstructure.


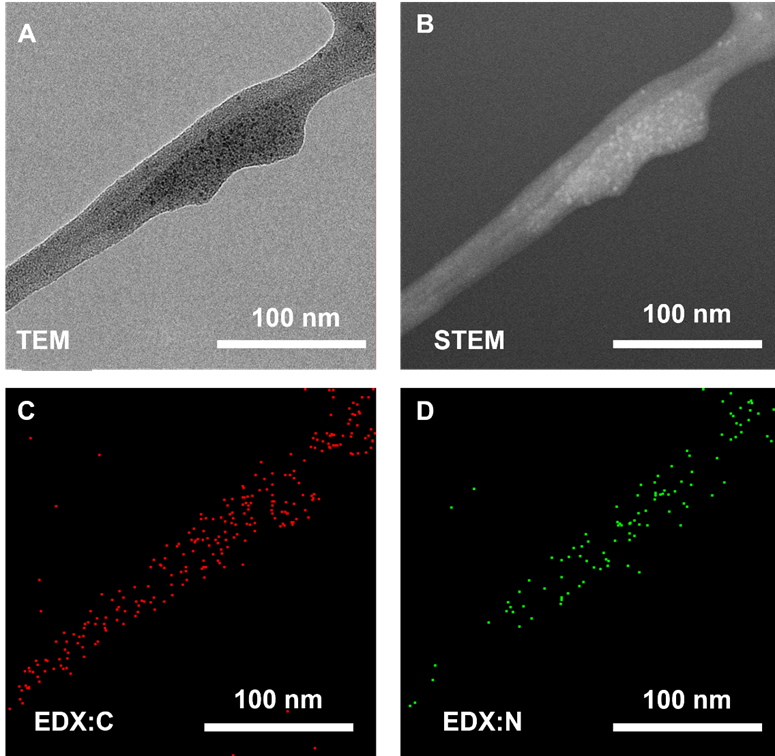


**Figure S15.** (A) TEM image of the Glu-CDs-BC. (B) STEM image of the Glu-CDs-BC. STEM-EDX mapping images of (C) the distribution of C and (D) the distribution of N.
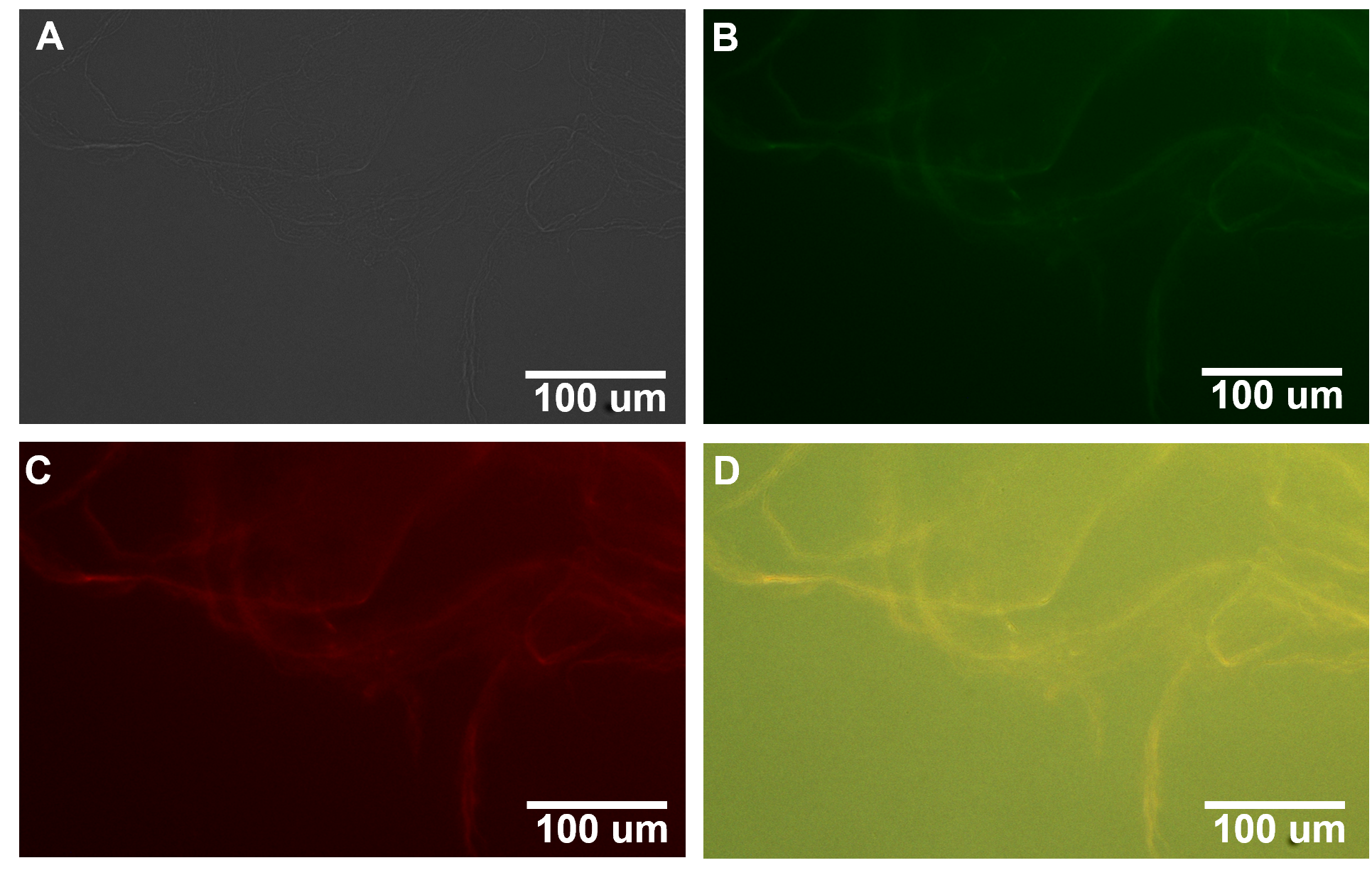


**Figure S16**. Images of Glu-CDs/BC before purification under fluorescence microscopy. (A) Bright-field image of Glu-CDs/BC before purification. (B) Fluorescence image of Glu-CDs/BC before purification under blue light excitation. (C) Fluorescence image of Glu-CDs/BC before purification under green light excitation. (D) A merged image of the images (A), (B), and (C).


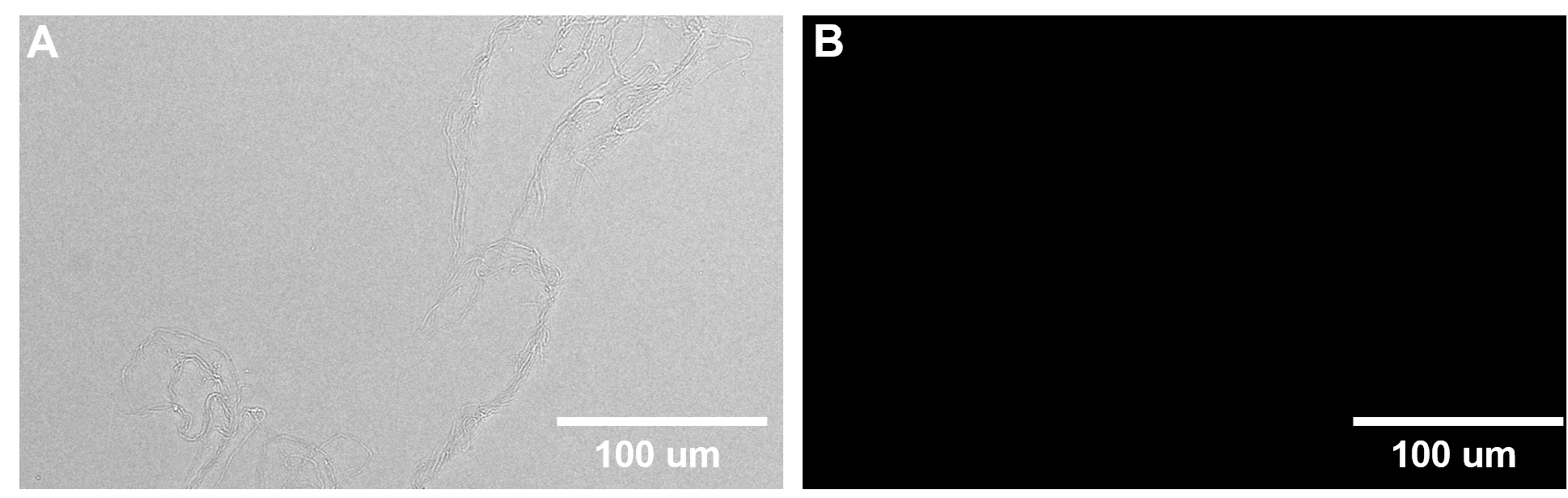


**Figure S17**. Images of Glu-CDs/BC after purification under fluorescence microscopy. (A) Bright-field image of Glu-CDs/BC after purification. (B) Fluorescence image of Glu-CDs/BC after purification under blue light excitation.

The fluorescence images of Glu-CDs/BC were obtained by using an inverted fluorescence microscope. Before purification, the surface of Glu-CDs/BC exhibited uniform fluorescence (**Figure S16**). However, after purification, Glu-CDs/BC did not exhibit any fluorescence (**Figure S17**).


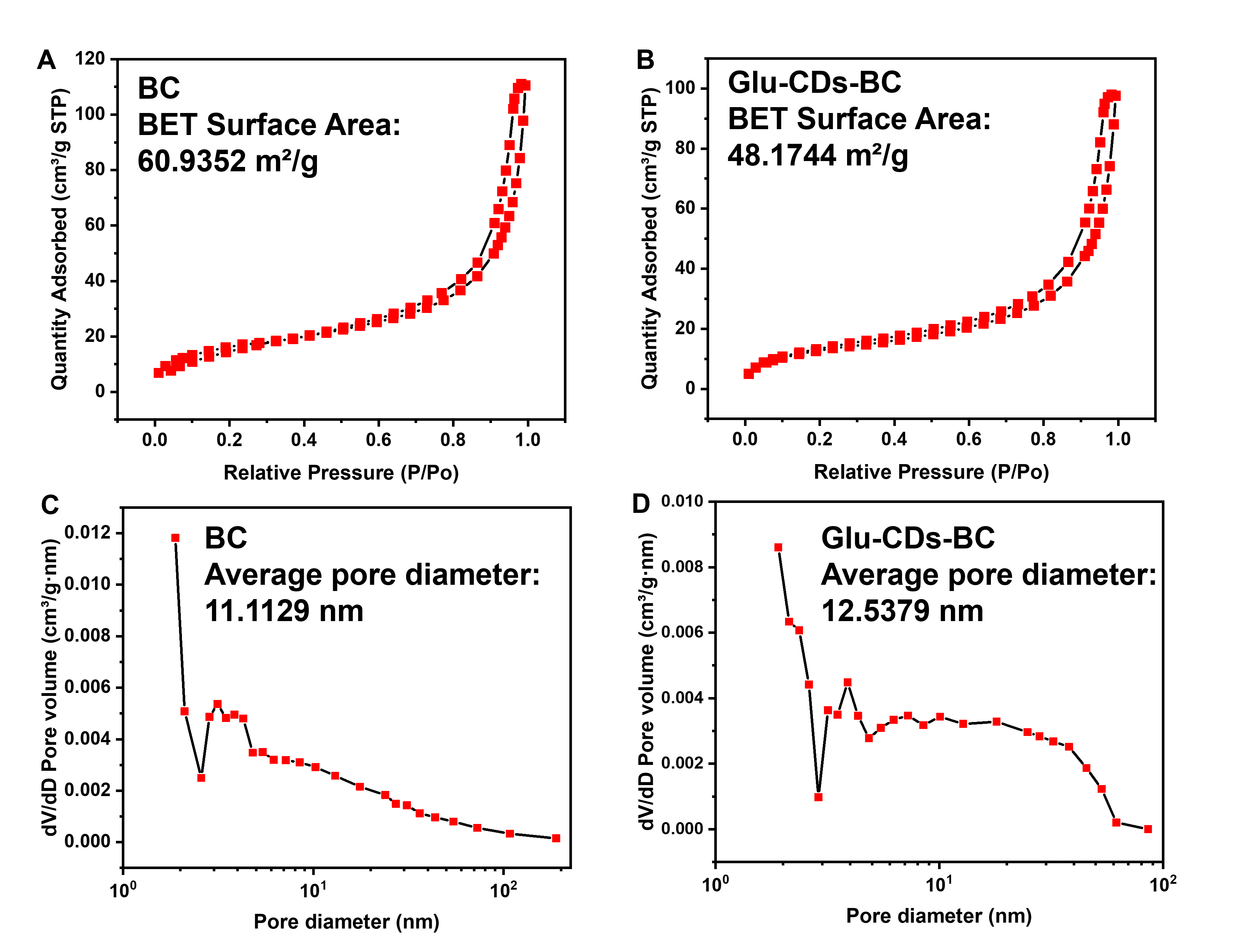


**Figure S18.** The N_2_ adsorption-desorption isotherms of (A) BC and (B) Glu-CDs-BC at 77 K. The pore size distribution of (C) BC and (D) Glu-CDs-BC at 77 K which was obtained from the Barret-Joyner-Halenda (BJH) model.


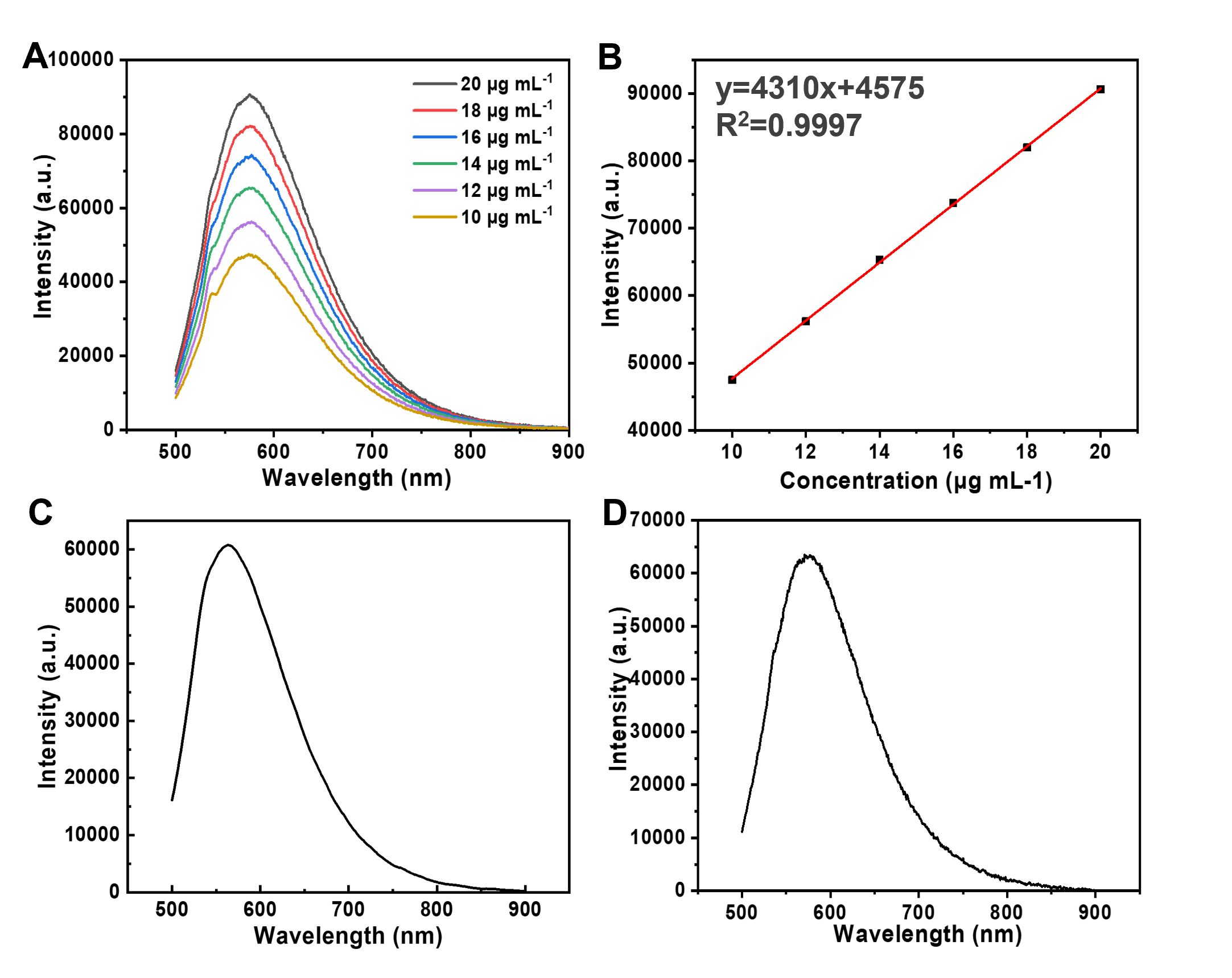


**Figure S19**. The contents of Glu-CDs in Glu-CDs/BC and Glu-CDs-BC. (A) Fluorescence emission spectra of Glu-CDs with different concentrations when excited at 480 nm. (B) The linearity of calibration correlation between the concentration of Glu-CDs and the emissive fluorescence intensity at 570 nm. (C) Fluorescence emission spectrum of the Glu-CDs dispersion separated and purified after preparing Glu-CDs-BC. (D) Fluorescence emission spectrum of the Glu-CDs dispersion separated from preparing Glu-CDs/BC.

The Glu-CDs were dispersed in water to form dilute suspensions with concentrations of 10 μg mL^−1^, 12 μg mL^−1^, 14 μg mL^−1^, 16 μg mL^−1^, 18 μg mL^−1^ and 20 μg mL^−1^, respectively. The emission spectra of these suspensions were measured under 480 nm excitation light (**Figure S19A**). As shown in **Figure S19B**, the concentration of Glu-CDs and the fluorescence intensity at 570 nm was fitted very well as a linear relationship. This fitted linear equation will be used as a standard curve to accurately determine the concentration of Glu-CDs. The *K. sucrofermentans* was cultured with sterilized HS basic medium supplemented with Glu-CDs (1 mg mL^−1^) at 30°C for 7 days. The resulting Glu-CDs-BC was removed from the culture medium, and then was treated with lysozyme solution (0.2%, w/v, 5 mL) at 30°C for 2 h. After this treatment, it was thoroughly washed with Milli-Q water thoroughly until no fluorescence was detected in the residual water. The remaining culture medium was treated with an ultrasonic dismembrator (Scientz-IID, China) at 100 W for 5 minutes to break the cell membranes of *K. sucrofermentans* and release the Glu-CDs inside the cells into the suspension. The water collected from washing the Glu-CDs-BC was combined with the culture medium after ultrasonic treatment, and more deionized water was added to make a total volume to 50 mL. A 2 mL portion of this 50 mL suspension was subjected to dialysis for 48 hours, with the water being changed every 12 hours. After dialysis, the liquid volume increased to 3.4 mL, and more deionized water was added to reach a total volume of 15 mL. The emission spectrum of the final suspension was then measured using a fluorescence spectrophotometer (**Figure S19C**). Based on the established linear equation in **Figure S19B**, the concentration of Glu-CDs in the suspension was calculated to be 13.04 µg mL^−1^. Consequently, the content of Glu-CDs in Glu-CDs-BC was determined to be 0.61%, using the following equation:

$$\omega_{1}=\frac{m-c_{1}\times V_{1}\times7.5}{m_{1}}\times100\%$$

where *m* stands for the mass of Glu-CDs in the initial suspension (5 mg); *c_1_* stands for the concentration of Glu-CDs (13.04 µg mL^−1^); *V_1_* stands for the volume of the suspension collected after separation and purification of Glu-CDs-BC (50 mL); *m*_1_ stands for the mass of Glu-CDs-BC (18.2 mg).

The BC was immersed in a 5 mL suspension of Glu-CDs (1 mg mL^−1^) at 30°C for 7 days. Afterward, the BC was removed from the Glu-CDs suspension, and the resulting material was labeled as Glu-CDs/BC. After the separation of Glu-CDs/BC, the remaining Glu-CDs suspension was diluted 50 times, then, its fluorescence emission spectrum was obtained using a fluorescence spectrophotometer (**Figure S19D**). Using this established linear equation in **Figure S19B**, the concentration of Glu-CDs in the suspension was calculated to be 13.67 µg mL^−1^. Consequently, the content of Glu-CDs in Glu-CDs/BC was determined to be 6.82%, using the following equation:

$$\omega_{2}=\frac{m-c_{2}\times V_{2}\times50}{m_{2}}\times100\%$$

where *m* stands for the mass of Glu-CDs in the initial suspension (5 mg); *c_2_* stands for the concentration of Glu-CDs (13.67 µg mL^−1^); *V_2_* stands for the volume of the suspension separated from preparing Glu-CDs/BC (5 mL); *m*_2_ stands for the quality of Glu-CDs/BC (23.2 mg).


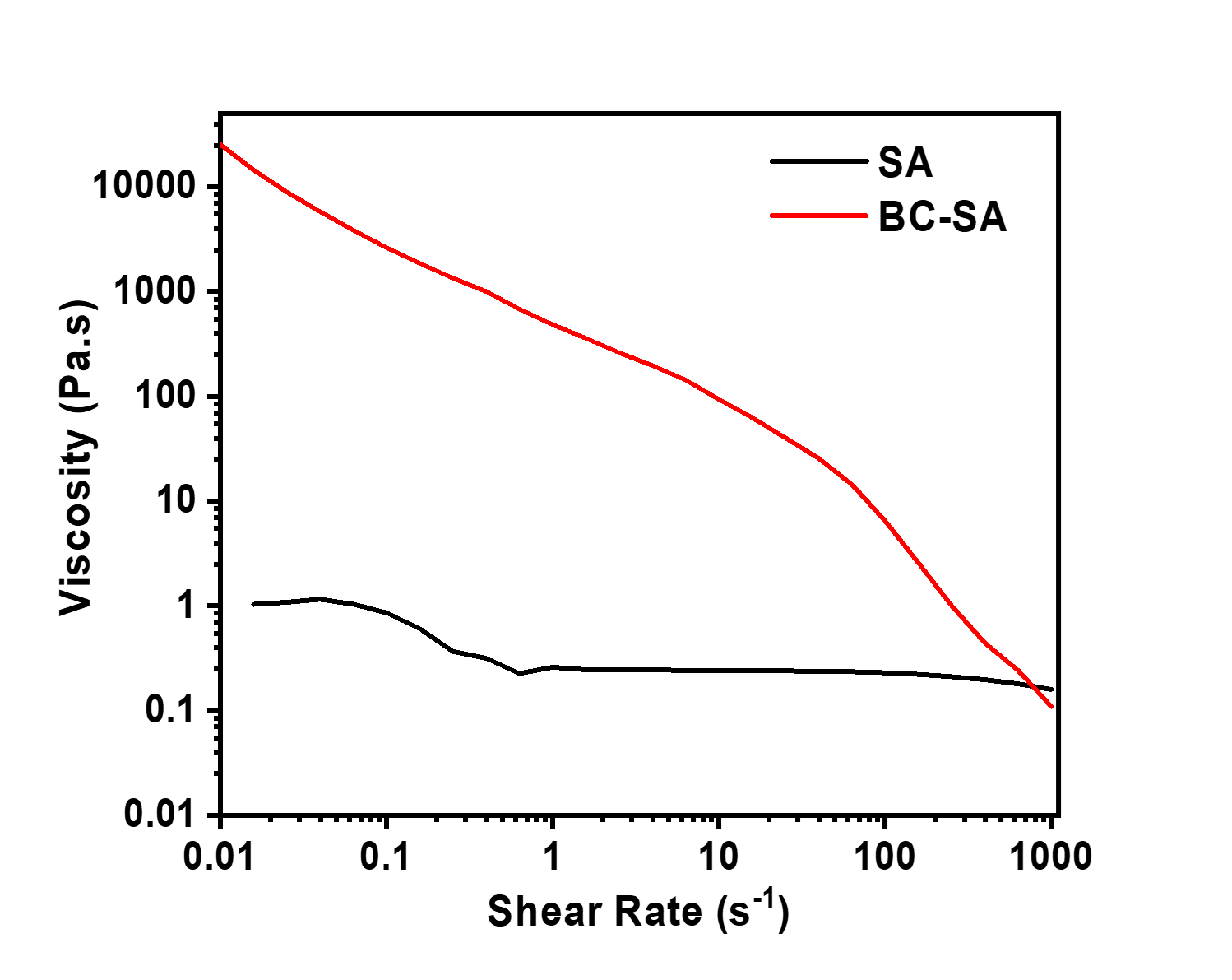


**Figure S20**. The flow curves of SA and BC-SA obtained for shear rates ranging from 0.01 to 1000 s⁻¹.

The rheological properties of the Glu-CDs-BC-SA hydrogel were thoroughly investigated, as shown in **Figure S20**. At low shear rates, Glu-CDs-BC-SA displayed exceptionally high viscosity (27500 Pa·s), significantly higher than that of pure SA. This high viscosity provided robust structural support, enabling the formation of stable 3D structures without collapse. At high shear rates (1000 s⁻¹), the viscosity of Glu-CDs-BC-SA decreased below that of pure SA, facilitating smooth extrusion and ensuring excellent printability.


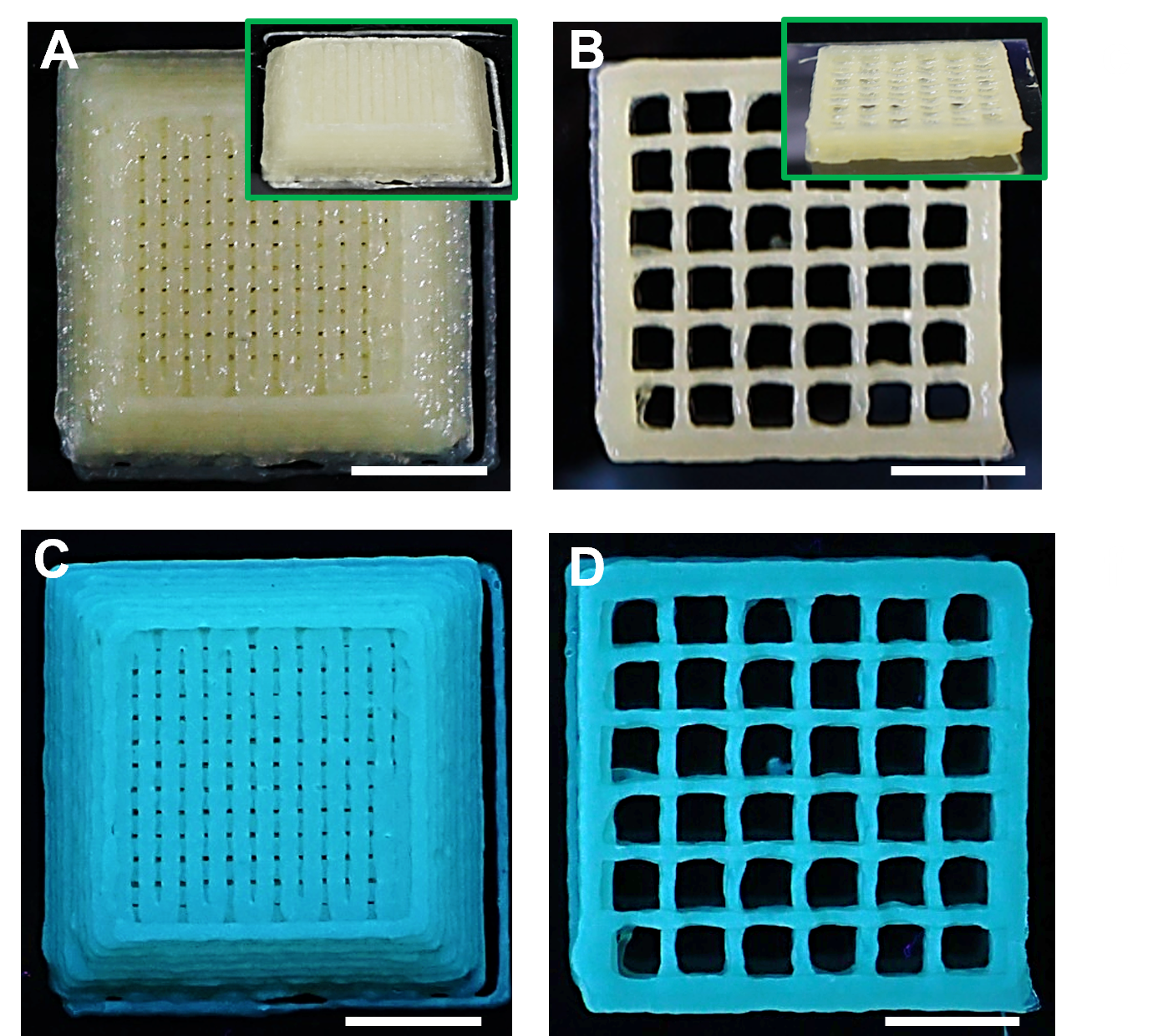


**Figure S21**. The photographs of various 3D geometric structures fabricated via 3D printing using Glu-CDs-BC-SA hydrogel, including (A) square frustum, (B) hollow cuboid. The Corresponding fluorescence images of these structures under 365 nm UV illumination, including (C) square frustum, (D) hollow cuboid. The scale bars in (A)–(D) is 1 cm.

Table S1. Yield, inoculum concentration, and thickness of BC and Glu-CDs-BC

|  | Glu-CDs | *K. sucrofermentans* concentration | Thickness (cm) | yield (mg/mL) |
| --- | --- | --- | --- | --- |
| BC | 0 | 10⁷-10^8^ CFU/mL | 0.37 | 6.7 |
| Glu-CDs-BC | 1 mg mL^−1^ | 10⁷-10^8^ CFU/mL | 0.31 | 5.5 |

Table S2. The yield and loading rate of Glu-CDs-BC obtained from culture media containing different concentrations of Glu-CDs

| Concentration of Glu-CDs (mg mL^−1^) | 0 | 0.5 | 1 | 1.5 | 2 |
| --- | --- | --- | --- | --- | --- |
| Glu-CDs loading capacity | 0 | 0.53% | 0.61% | 0.67% | 0.71% |
| Glu-CDs-BC yield (mg/mL) | 6.7 | 6.1 | 5.5 | 3.2 | 0.81 |

**Reference**

(1) Schneider, J.; Reckmeier, C. J.; Xiong, Y.; von Seckendorff, M.; Susha, A. S.; Kasák, P.; Rogach, A. L. Molecular Fluorescence in Citric Acid-Based Carbon Dots. *J. Phys. Chem. C* **2017**, *121* (3), 2014-2022. DOI: 10.1021/acs.jpcc.6b12519.
